# The Gut Microbiome Dynamically Associates with Host Glucose Metabolism throughout Pregnancy: Longitudinal Findings from a Matched Case‐Control Study of Gestational Diabetes Mellitus

**DOI:** 10.1002/advs.202205289

**Published:** 2023-01-22

**Authors:** Zhonghan Sun, Xiong‐Fei Pan, Xiao Li, Limiao Jiang, Ping Hu, Yi Wang, Yi Ye, Ping Wu, Bin Zhao, Jianguo Xu, Mengmeng Kong, Yanni Pu, Manying Zhao, Jianying Hu, Jinfeng Wang, Guo‐Chong Chen, Changzheng Yuan, Yongfu Yu, Xiang Gao, Fangqing Zhao, An Pan, Yan Zheng

**Affiliations:** ^1^ State Key Laboratory of Genetic Engineering School of Life Sciences and Human Phenome Institute Fudan University Shanghai China; ^2^ Ministry of Education Key Laboratory of Contemporary Anthropology Fudan University Shanghai China; ^3^ Section of Epidemiology and Population Health Ministry of Education Key Laboratory of Birth Defects and Related Diseases of Women and Children West China Second University Hospital & West China Biomedical Big Data Center West China Hospital Sichuan University; Shuangliu Institute of Women's and Children's Health Shuangliu Maternal and Child Health Hospital Chengdu Sichuan China; ^4^ Department of Epidemiology & Biostatistics School of Public Health Tongji Medical College Huazhong University of Science and Technology Wuhan Hubei China; ^5^ Key Laboratory of Environment & Health (Huazhong University of Science and Technology) Ministry of Education Wuhan Hubei China; ^6^ Antenatal Care Clinics Shuangliu Maternal and Child Health Hospital Chengdu China; ^7^ Department of Clinical Laboratories Shuangliu Maternal and Child Health Hospital Chengdu China; ^8^ Beijing Institutes of Life Science Chinese Academy of Sciences Beijing China; ^9^ Department of Nutrition and Food Hygiene School of Public Health Soochow University Suzhou China; ^10^ School of Public Health Zhejiang University School of Medicine Hangzhou Zhejiang China; ^11^ School of Public Health Fudan University Shanghai China

**Keywords:** gestational diabetes mellitus, glucose metabolism, gut microbiome, matched case‐control study

## Abstract

Though gut microbiome disturbance may be involved in the etiology of gestational diabetes mellitus (GDM), data on the gut microbiome's dynamic change during pregnancy and associations with gestational glucose metabolism are still inadequate. In this prospective study comprising 120 pairs of GDM patients and matched pregnant controls, a decrease in the diversity of gut microbial species and changes in the microbial community composition with advancing gestation are found in controls, while no such trends are observed in GDM patients. Multivariable analysis identifies 10 GDM‐related species (e.g., *Alistipes putredinis*), and the integrated associations of these species with glycemic traits are modified by habitual intake of fiber‐rich plant foods. In addition, the microbial metabolic potentials related to fiber fermentation (e.g., mannan degradation pathways) and their key enzymes consistently emerge as associated with both GDM status and glycemic traits. Microbial features especially those involved in fiber fermentation, provide an incremental predictive value in a prediction model with established risk factors of GDM. These data suggest that the gut microbiome remodeling with advancing gestation is different in GDM patients compared with controls, and dietary fiber fermentation contributes to the influence of gut microbiome on gestational glycemic regulation.

## Graphical abstract

The temporal remodeling in gut microbiota during pregnancy is related to the development of gestational diabetes mellitus (GDM). Habitual intake of fiber‐rich foods modifies the associations of microbial species with glycemic traits during pregnancy. Microbial features measured at early pregnancy provides an incremental predictive value when added to traditional predictive model for GDM risk.

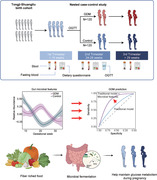

## 1 Introduction

Gestational diabetes mellitus (GDM), one of the major pregnancy complications, may result in multiple disorders and diseases for mothers and their offspring through a transgenerational flow.^[^ ^1^, ^2^ ^]^ A prior history of GDM could lead to a 10‐fold increased risk of type 2 diabetes mellitus (T2DM) for mothers and an 8‐fold higher risk of pre‐diabetes for their offspring.^[^ ^3^, ^4^, ^5^ ^]^ The metabolic modifications during pregnancy prompt fetal development, while the maladaptation of these modifications may have a diabetogenic effect on maternal metabolism and lead to the development of GDM. However, the underlying complex system of gestational adaptation or maladaptation remains inadequately explored.

The gut microbiome has been implicated in GDM pathogenesis.^[^ ^6^, ^7^ ^]^ Compared to those with normal glycemic status, an altered composition of the gut microbiota has been found in GDM patients.^[^ ^8^, ^9^, ^10^, ^11^ ^]^ However, previous studies failed to characterize the dynamic remodeling process of the gut microbiome during pregnancy due to single‐sampling strategy. Hence, the longitudinal associations of the gut microbiome with gestational glucose metabolism are unclear. And most of the published results were generated using 16S rRNA gene profiles, which lacked microbial taxonomic and functional resolution. Furthermore, whether diet could influence microbial associations with host glucose metabolism during pregnancy remains to be explored.

**Figure 1:**
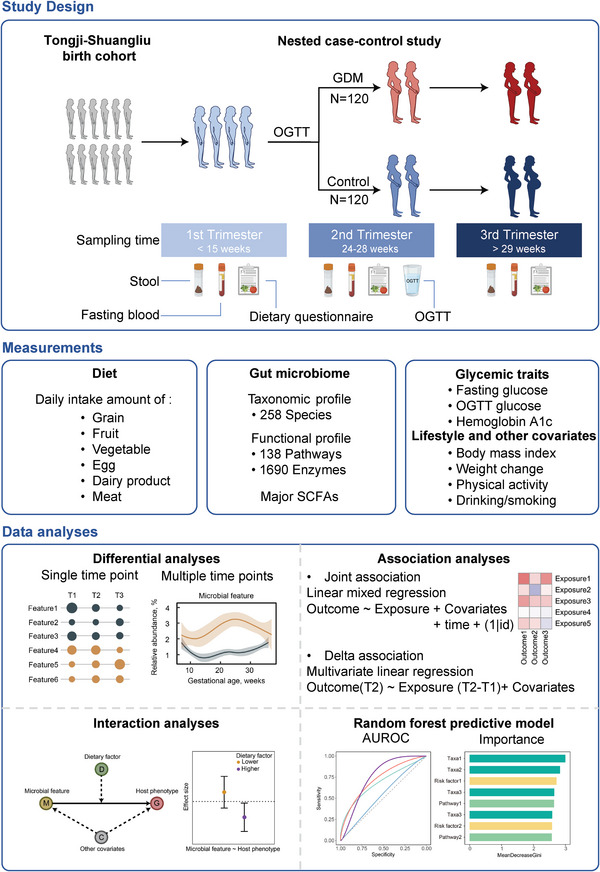
Study design, measurements, and analysis strategy. To associate the gut microbiome with diet and glucose metabolism, we profiled stool metagenomes and glycemic traits from a prospective case‐control study nested in the Tongji‐Shuangliu birth cohort. Blood and stool samples, dietary records, and health‐related information were collected, and taxonomic and functional profiling from stool shotgun metagenomes, fecal short chain fatty acids (SCFAs), plasma biomarkers of glucose metabolism, and other covariates were measured at each trimester (n = 720). The joint associations between microbial features and glycemic traits were estimated using linear mixed models with pooled data from three trimesters (n = 720). The delta associations were estimated using the change values of microbial features between the first trimester (T1) and the second trimester (T2) and oral glucose tolerance test (OGTT) measured in T2 (n = 240). Interaction analyses were performed to explore the potential interaction effect of dietary factors on the associations between microbial features and host glucose metabolism. Random forest classification models were constructed to estimate the prediction power of microbial data for the risk of gestational diabetes mellitus (GDM). The area under operating characteristic curve (AUROC) was used as a metric to quantify classifiers performance. Icons representing the types of collected samples were created using Biorender.com.

In this prospective study, 120 pairs of GDM patients and matched healthy pregnant controls were included, and their comprehensive clinical measurements, habitual dietary intakes, and metagenomic profile of gut microbiome were recorded at each trimester (T) (**Figure** **1**), in order to achieve the objectives of: 1) describing the longitudinal adaptations in gut bacterial taxonomic and functional features during pregnancy in women with and without GDM; 2) estimating the dynamic associations of the microbial features with host glucose metabolism during pregnancy; and 3) exploring whether and to what extent habitual food consumption could modulate such associations.

## 2 Results and Discussion

### 2.1 Characteristics of the Study Participants during Pregnancy

A total of 120 pairs of GDM patients and matched controls from the Tongji‐Shuangliu Birth Cohort (TSBC) were included in this study (Figure 1). The mean (standard deviation) age at enrollment of the participants was 27.4 (3.9) years. No difference was observed in education level, smoking status, alcohol consumption, or family history of T2DM between the GDM patients and controls (Table S1, Supporting Information). Compared with controls, GDM patients had a higher mean BMI (body mass index) in pre‐pregnancy, T1, and T2 and a higher mean gestational weight gain (GWG) in T3 (all *P* < 0.05). Though not statistically significant, GDM patients showed a trend to have a higher mean GWG than controls (1.2 vs 0.7 kg, *P* = 0.18). In addition, GDM patients were more likely to have higher levels of fasting plasma glucose (FPG) and hemoglobin A1c (HbA1c) compared with controls, though most of these differences (except for HbA1c) were weakened in T3 (all *P* > 0.05, Table S1, Supporting Information).

### 2.2 Different Adaptations of Microbial Composition During Pregnancy in GDM Patients

**Figure 2:**
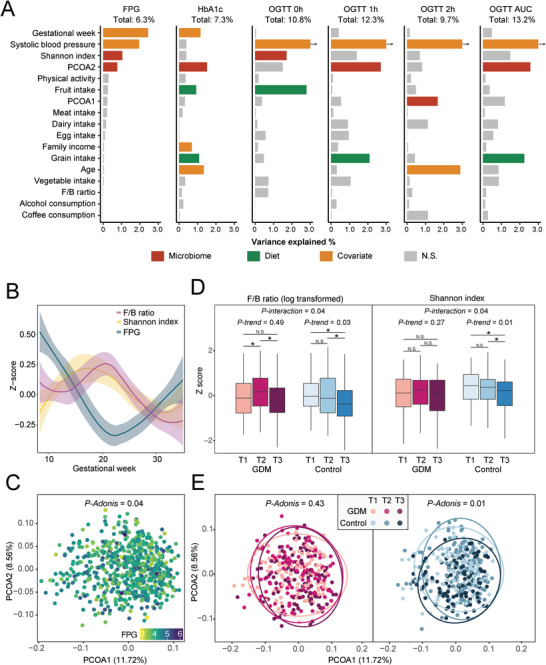
The gut microbiome composition among GDM patients and controls. A) The variance of glycemic traits explained by gut microbial composition, diet, and other covariates. The height of each bar represented the explained variance calculated using univariate linear regression. The color bar represented significant associations between glycemic traits and host factors (*P* < 0.05), while the grey bars represented non‐significant ones. The explained variances of fasting plasma glucose (FPG) and hemoglobin A1c (HbA1c) were estimated with pooled data from three trimesters, while the explained variances of OGTT glucose were estimated with data collected at T2. B) Distributions of Firmicutes to Bacteroidetes (F/B) ratio, microbial *α*‐diversity, and host FPG among all participants during pregnancy. The microbial *α*‐diversity was represented by the Shannon index. C) Principal coordinate analysis of the gut microbiome of all samples was conducted using species‐level Bray‐Curtis distance. The color gradient of dots represented matched host FPG. D) The temporal change of microbial F/B ratio (left) and *α*‐diversity (right) among participants with GDM and controls. The boxes in red represented samples from women with GDM and those in blue represented samples from controls. The inter‐group differences in F/B ratio and *α*‐diversity at each trimester were tested using the student's t‐test. The intra‐group change trends in F/B ratio and *α*‐diversity between trimesters were tested using linear regression. An interaction term of GDM status and trimester was further included to evaluate the effect of GDM status on these temporal changes. E) The temporal change of gut microbial composition among women with GDM (left) and controls (right) during the pregnancy. The intra‐group differences in *β*‐diversity between trimesters were calculated using permutational multivariate analysis of variance (PERMANOVA).

Overall, the gut microbial *α*‐diversity (i.e., Shannon index) and microbial composition (i.e., eigenvalues of the principal coordinates analysis) explained a significant amount of variation in gestational glycemic traits (e.g., 1.6% of the variance in FPG, 2.1% of the variance in HbA1c), which was comparable to that of established clinical risk factors (e.g., gestational week and systolic blood pressure, **Figure** **2**A).

During pregnancy, an inverse association of Firmicutes to Bacteroidetes (F/B) ratio and microbial *α*‐diversity (i.e., Shannon index) with host FPG was observed (both *P* < 0.05, Figure 2B). Furthermore, the overall composition of gut microbiome was also associated with FPG (*P* = 0.04, Figure 2C). With advancing gestation, decreasing trends in the F/B ratio and microbial *α*‐diversity were observed among controls (both *P‐trend* < 0.05, Figure 2D and Figure S1, Supporting Information) but not in GDM patients (both *P‐interaction* of GDM < 0.05, Figure 2D). Similarly, time‐dependent alterations in general microbial composition were observed in controls but not in GDM patients (Figure 2E). Compared with controls, GDM patients tended to have a reduced gut microbiome diversity in T1 (*P* = 0.04, Figure S2A, Supporting Information), while the differences in microbial composition were consistently observed in T2 and T3 (both *P* < 0.05, Figure S2B, Supporting Information).

### 2.3 Diet Modified the Associations between Species and Host Glucose Metabolism

**Figure 3:**
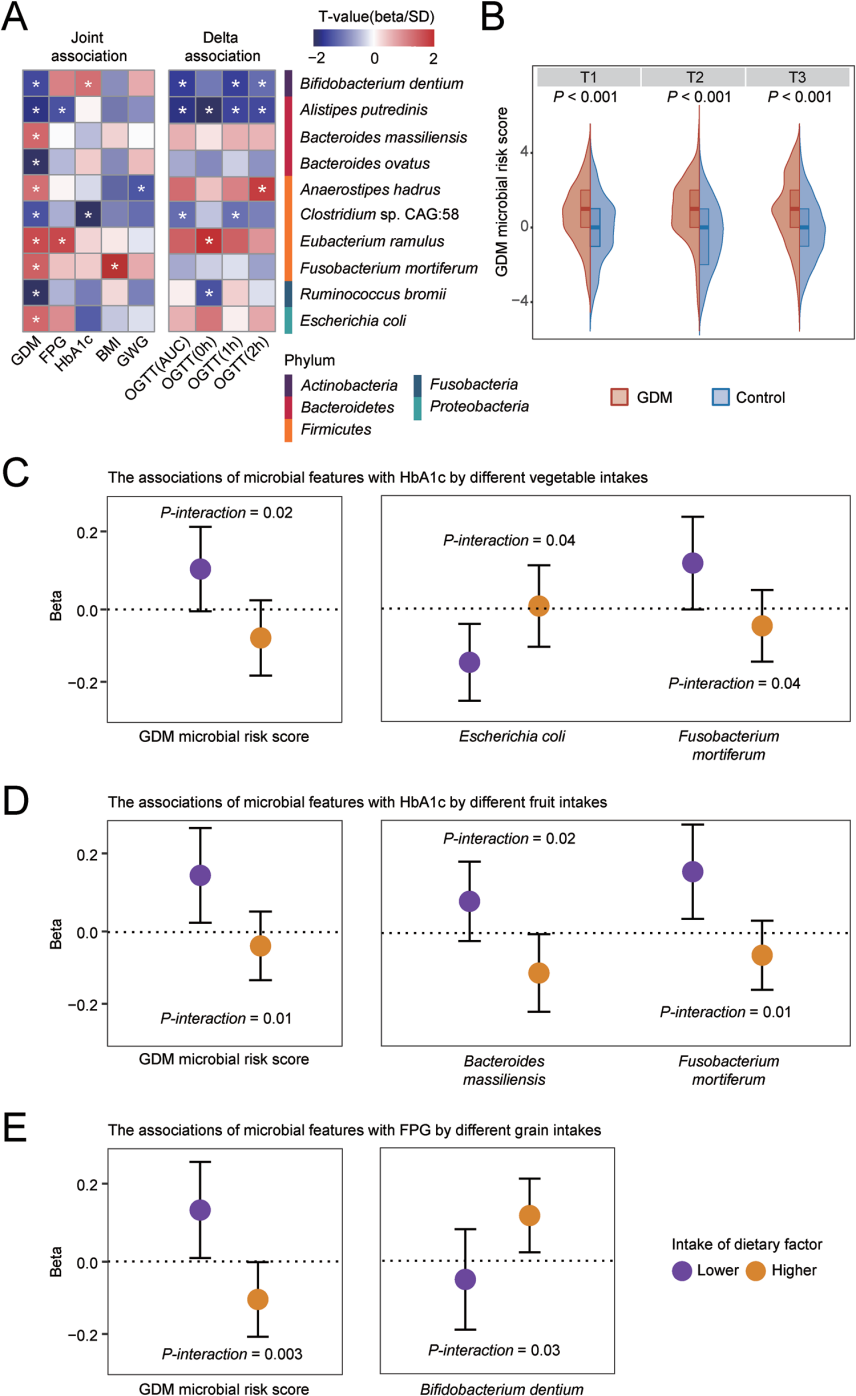
The associations of GDM microbial risk score and species with host glucose metabolism during pregnancy. A) The associations of GDM‐related species with host glucose metabolism and body weight. Asterisks represented FDR‐corrected *P* <0.25. B) The distribution of GDM microbial risk score in GDM patients and control at each trimester. C–D) The association between microbial features and HbA1c was modified by vegetable intake (C) and fruit intake (D). E) The association between microbial features and FPG was modified by grain intake.

Among the 258 analyzed microbial species, 10 species from five phyla were identified to be associated with GDM status in the adjusted linear mixed models (joint association, false discovery rate (FDR) corrected *P* < 0.25, **Figure** **3**A), of which five species were enriched in controls compared with GDM patients during pregnancy (Figure S3, Supporting Information). For example, compared with controls, a consistently lower relative abundance of *Ruminococcus bromii* was observed in GDM patients throughout three trimesters; *Alistipes putredinis* and *Bacteroides ovatus* remained lower in T1 and T2 as well (FDR‐corrected *P* < 0.25, Table S2, Supporting Information). Among these GDM‐related species, six species were further associated with host glycemic traits, BMI, or GWG during pregnancy (joint association, FDR‐corrected *P* < 0.25, Figure 3A). For example, the relative abundance of *A. putredinis* was inversely associated with FPG (*β* = −0.01) and *Eubacterium ramulus* was positively associated with FPG (*β* = 0.003). These two species’ changes between T1 and T2 were further associated with the glucose measurements during the oral glucose tolerance test (OGTT) in T2 (delta association, FDR‐corrected *P* < 0.25, Figure 3A).

A composite microbial risk score calculated based on the presence of these 10 GDM‐related species (see Experimental Section) was consistently higher in GDM patients compared with controls at each trimester as expected (*P* < 0.001, Figure 3B). Of note, the associations between this GDM microbial risk score and glycemic traits were significantly modified by habitual intake of fiber‐rich plant foods. For example, positive associations of the GDM microbial risk score with HbA1c or FPG were only observed among participants with a lower intake of fruits, vegetables, and grains (*P‐interaction* < 0.05, Figure 3C–E). Similar modification effects of the fiber‐rich plant foods were also observed among some component microbial species, including *Escherichia coli*, *Fusobacterium mortiferum*, *Bacteroides massiliensis*, and *Bifidobacterium dentium* (Figure 3C–E).

### 2.4 Microbial Fermentation was Associated with Host GDM Status and Glycemic Metabolism

**Figure 4:**
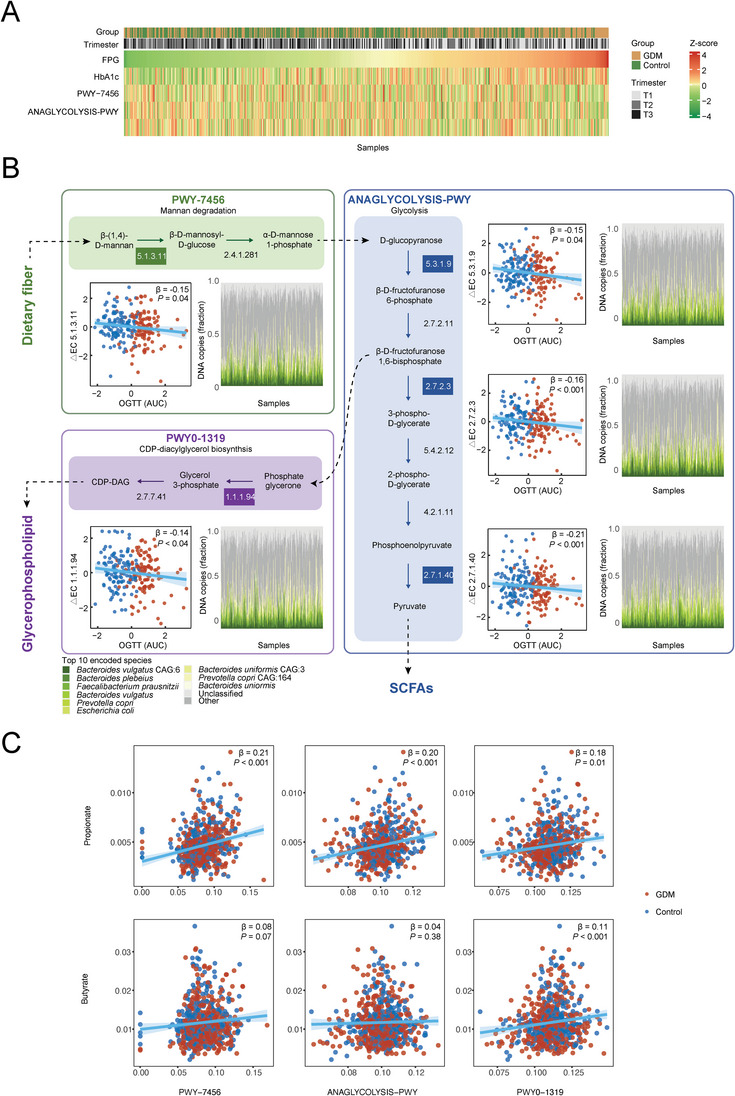
A) The associations of microbial fiber fermentation related pathways with host glucose metabolism during pregnancy (Revised part). B)The associations of changes in key enzymes from T1 to T2 within the pathways of polysaccharide degradation (green), glycolysis (blue), and phospholipid biosynthesis (purple) with host OGTT glucose. The scatter plots showed the associations of these enzymes’ temporal change (from T1 to T2) with the area under OGTT glucose curve in T2. Red and blue dots indicated GDM patients and controls, respectively. The stack plots showed the proportions of enzymes encoded by specific species, whose colors were shown in the top legend. C) The associations of fermentation pathways and fecal levels of propionate and butyrate.

With multivariable adjustment for covariates, 26 microbial functional pathways had different abundances between GDM cases and controls (joint association, FDR‐corrected *P* < 0.25, Figure S4, Supporting Information). Generally, the gut microbiome of GDM patients showed a decreased capacity for fermentation, biosynthesis of lipids, and nucleotides in T1 but an increased capacity for degradation of carbohydrates and nucleotides in T2 and T3 (FDR‐corrected *P* < 0.25, Figure S4 and Table S3, Supporting Information). Furthermore, among 13 GDM‐related microbial pathways involved in the microbial metabolism of carbohydrates, significant associations were identified with glycemic traits, BMI, or GWG during pregnancy (joint association, FDR‐corrected *P* <0.25, Figure S4A, Supporting Information). For example, the mannan degradation pathway (PWY‐7456) was inversely associated with FPG, HbA1c, BMI, and GWG (all FDR‐corrected *P* < 0.25, Figure S4A, Supporting Information). Mannan, an insoluble fiber embedded in almost all plant cell walls, has been found to suppress lipid accumulation through microbial degradation to short‐chain fatty acids (SCFAs).^[^ ^12^ ^]^ According to previous studies, the mannan degradation pathway (PWY‐7456) was enriched in people who adhered to the Mediterranean Diet, which is a healthy plant‐based dietary pattern.^[^ ^13^ ^]^ Notably, two downstream pathways of mannan degradation (i.e., glycolysis [ANAGLYCOLYSIS‐PWY] and CDP‐diacylglycerol biosynthesis [PWY0‐1319]) also exhibited inverse associations with FPG levels (FDR‐corrected *P* < 0.25, **Figure** **4**A and Figure S4A, Supporting Information). Through mannan degradation, the gut bacteria produce D‐glucopyranose and thus provide substrates for the downstream microbial glycolysis, fermentation, and phospholipid biosynthesis (Figure 4B). In addition, OGTT results showed a significant inverse association with the key enzymes in these pathways related to fiber fermentation, such as cytosolic pyruvate kinase (EC 2.7.1.40), which is a key enzyme in the synthesis of pyruvate and ATP, and glycerol‐3‐phosphate dehydrogenase (EC 1.1.1.94), which is a key enzyme connecting carbohydrate and lipid metabolism (Figure 4B).^[^ ^14^, ^15^ ^]^ These enzymes were mainly encoded by SCFA‐producing bacterial species, including *Bacteroides vulgatus*, *Bacteroides plebeius*, and *Faecalibacterium prausnitzii* (Figure 4B).^[^ ^16^, ^17^ ^]^ Notably, *Ba. plebeius* ranked second in total contribution to these enzymes, which was more likely to have a lower relative abundance in GDM patients compared with controls in T1 (Table S2, Supporting Information).

### 2.5 Temporal Changes of Fecal SCFAs were Different in Participants with GDM

To further explore the potential mechanisms underlying the association between gut microbiome and GDM, fecal levels of major SCFAs (i.e., acetate, propionate, and butyrate) were measured at each trimester. Although there were no significant differences in the fecal levels of major SCFAs between GDM patients and controls at each trimester (all *P* > 0.05), the temporal increase of propionate during T1 and T2 was greater in controls than that in GDM patients (*P* < 0.05, Figure S5, Supporting Information). Furthermore, the temporal changes in fecal levels of propionate and butyrate during T1 and T2 were inversely associated with plasma glucose levels during OGTT in T2 (all *P* < 0.05, Figure S6, Supporting Information). In addition, fecal levels of propionate were positively associated with aforementioned fermentation‐related pathways (i.e., PWY−7456, ANAGLYCOLYSIS‐PWY, and PWY0‐1319, all *P* < 0.05), and fecal levels of butyrate showed a similar trend of such associations though only their association of PWY0‐1319 was significant (Figure 4C).

### 2.6 Microbial Features in Early Pregnancy Improved the Prediction of GDM

**Figure 5:**
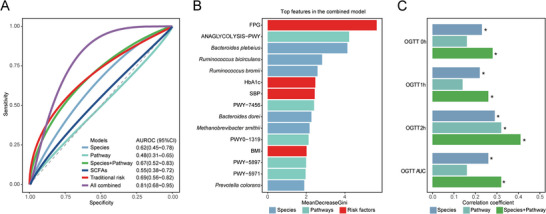
Performance of the random forest predictive models of GDM and OGTT glucose based on microbial features. A) Random forest classification models of GDM based on microbial features and traditional risk factors in T1. The predictive power of classification models was shown by the area under the receiver operating characteristic curve. The colors of the curves represented the data used to generate the predictive model. B) The most important factors in the combined prediction model for GDM. The width of each bar represented the importance of the corresponding variable, estimated by the mean decrease in the Gini index of random forest models. C) Random forest regression models of OGTT glucose based on microbial features. The predictive power of regression models was shown by the correlation between predicted values and observed values. Asterisks represented *P* < 0.05.

At early pregnancy (T1), the model of microbial species performed better in predicting the risk of GDM compared to the model of microbial functional pathways (area under the receiver operating characteristic curve [AUROC] for species model [95% CI]: 0.62 [0.45–0.78], and that for pathway model: 0.48 [0.31–0.65], *P‐difference* = 0.04, **Figure** **5**A). An addition of microbial features into a comprehensive well‐known model including traditional risk factors (see Experimental Section, AUROC: 0.69 [0.56–0.82]) significantly improved the prediction performance (AUROC of the combined model: 0.81 [0.68–0.95], *P* = 0.01, Figure 5A). The aforementioned pathways related to fiber fermentation (i.e., glycolysis, mannan degradation, and CDP‐diacylglycerol biosynthesis) and *Ba. plebeius* (a carrier of key enzymes in fiber fermentation) were all listed among the most important features in the combined model (Figure 5B). Furthermore, random forest regression showed that microbial features in T1 were able to predict the OGTT glucose in T2, especially in the models with both microbial species and functional pathways (range of the Pearson's correlation coefficient between predicted values and observed values: 0.28–0.41, all *P* < 0.05, Figure 5C).

### 2.7 Discussion

By integrating serial measurements across different trimesters, our study is the first to report different microbial remodeling patterns between GDM patients and controls during their pregnancy. We identified a series of microbial species and metabolic potentials associated with gestational glycemic dysregulation, especially those involved in fiber fermentation (e.g., *A. putredinis*, and pathways related to fiber fermentation), and found that GDM‐related microbial species’ associations with glycemic traits were modulated by habitual intakes of fiber‐rich foods. Furthermore, we found that the gut microbial features at early pregnancy significantly increased the predictive power of an empirical model for GDM risk.

Although previous studies have reported the gut microbial alterations in GDM, the temporal change in the microbiome during pregnancy and its association with the development of GDM remains unclear.^[^ ^11^, ^18^, ^19^ ^]^ In our study, the composition of the gut microbiome altered before the diagnosis of GDM and remained so throughout pregnancy. Significant time‐dependent changes in microbial composition were observed in both GDM patients and controls; however, only in controls, the microbial diversity decreased with advancing gestation. Our observations were consistent with previous evidence, reporting the decreased microbial diversity with advancing gestation.^[^ ^20^, ^21^, ^22^ ^]^ This phenomenon might be due to the metabolic modifications occurred during pregnancy, including increased levels of blood glucose and hormone, which could change the internal environment of the human body.^[^ ^22^, ^23^ ^]^ In addition, the F/B ratio, a biomarker of gut microbial homeostasis,^[^ ^24^ ^]^ showed a different temporal trend among GDM patients compared to controls. By revealing changes in the temporal remodeling pattern of gut microbial composition in GDM patients, our study suggested that the gut microbiome might reflect the development and prognosis of GDM.

Our study identified a series of microbial signatures for GDM status. Five species were found to be enriched in GDM patients, among which *Bacteroides massiliensis* has been reported to be associated with the status of GDM, and *E. ramulus* and *Anaerostipes hadrus* were associated with an impaired glucose tolerance.^[^ ^25^, ^26^ ^]^ These GDM‐enriched species might participate in the development of GDM by influencing host immune status. For example, *B. massiliensis* could influence the expression of the RAC1 pathway and thus disturb gut epithelial homeostasis and host immune response.^[^ ^27^ ^]^ We also identified several species depleted in GDM patients, including *R. bromii* and *A. putredinis*, and *Bi. dentium*, which showed beneficial effects on human metabolic health.^[^ ^28^, ^29^, ^30^ ^]^ Consistent with our results, lower relative abundances of *R. bromii* and *A. putredinis* have been reported in GDM patients.^[^ ^19^, ^31^ ^]^ As a keystone species for starch degradation,^[^ ^32^ ^]^ *R. bromii* and *A. putredinis* could produce SCFAs, especially butyrate, and thus prevent inflammation and insulin resistance.^[^ ^33^, ^34^ ^]^ These species could be significantly influenced by diet, for example, the relatively lower dietary fiber and higher fat intakes among GDM patients might lead to their decreased abundances and thus further influence host glucose metabolism.^[^ ^35^, ^36^ ^]^ Together with previous findings, we identified several shared and specific microbial associations across different metabolic diseases. For example, the relative abundance of *A. putredinis* was inversely associated and that of *E. coli* was positively associated with the risk of both GDM and T2DM,^[^ ^29^, ^37^, ^38^ ^]^ while other species showed different associations between the two diseases. The association between *R. bromii* and host HbA1c level has been reported in T2DM,^[^ ^39^ ^]^ while no associations with host glycemic traits were observed in our study. The inconsistency in such microbe‐host interaction might be due to the unique physical adaptations during pregnancy other than insulin resistance, such as elevated hormones.^[^ ^1^ ^]^

Our data suggested that the fiber fermentation of gut microbiome might play a role in influencing the gestational glucose metabolism and development of GDM. We found that the intake of fiber‐rich foods could modify the associations of microbial features with host glucose metabolism and the microbial fiber fermentation capacity was inversely associated with gestational glucose. Our observations were supported by recent evidence that dietary fibers could alleviate T2DM development by modulating the gut microbiome.^[^ ^40^ ^]^ Therefore, the microbial fiber fermentation pathway may be beneficial to the glycemic regulation in both T2DM and GDM. In addition, we also observed a decreased metabolic capacity of gut microbiome to degrade dietary fiber among GDM patients and were inversely associated with gestational glucose. For example, the mannan degradation pathway (PWY‐7456), which could digest polysaccharides and provide substrates for the downstream microbial metabolism like glycolysis and phospholipids biosynthesis,^[^ ^41^ ^]^ had lower relative abundance among GDM patients and showed an inverse association with the FPG in our study. Consistent with our results, a recent study also reported the beneficial effect of PWY‐7456 on human health, which was driven by adherence to a Mediterranean diet rich in plant fiber.^[^ ^13^ ^]^ Notably, two downstream pathways of PWY‐7456 (i.e., ANAGLYCOLYSIS‐PWY and PWY0‐1319) and their key enzyme were also depleted in GDM patients and were associated with host glucose metabolism. The joint involvement of these pathways in the biosynthesis of SCFAs and glycerophospholipids might underlie the GDM pathophysiology through insulin resistance.^[^ ^42^, ^43^ ^]^ We also identified the positive associations between the aforementioned fiber‐related microbial features and major SCFAs, which could maintain the gut environment, mitigate inflammation, and regulate host glucose.^[^ ^44^ ^]^ These pieces of evidence jointly suggested that the gut microbiome could digest dietary fiber into SCFAs and thus help maintain normal glucose metabolism during pregnancy and prevent the development of GDM.

In the current study, the microbial features measured at early pregnancy showed robust predictive values in predicting GDM diagnosis in T2. When added to a well‐known clinical model of risk factors, they provided an incremental predictive value as well. As a non‐invasive diagnostic approach, the gut microbiome measurement at early pregnancy may help to identify the population at high risk, which is of great public health importance. However, further validation studies and experimental research are warranted for future clinical applications and implementation. Notably, since the microbial species and pathways involved in fiber fermentation were among the most important features contributing to the full prediction model, this finding further emphasized the crucial roles of dietary fiber's microbial fermentation in gestational glucose regulation.

To our knowledge, this is the largest study that has profiled the temporal change of the gut microbiome during pregnancy in participants with and without GDM. In addition, it is also the first study that demonstrated the importance of microbial fermentation of dietary fiber in maintaining normal glucose adaptation throughout pregnancy. These observations will contribute to the understanding of the interplay between the gut microbiome and gestational metabolic adaptation and provide new strategies for early GDM prevention as well as glucose management during pregnancy. With the longitudinal design, we closely observed the intra‐individual physiologic adaptation during pregnancy, which is predominant in the context of the gut microbiome.^[^ ^45^ ^]^ Nevertheless, our study also has limitations. First, because of the observational design, our study cannot make a causal inference. The specific impact on the gut microbiome from the general management of GDM could not be estimated because we did not have a direct measurement of this management. Despite adjusting for multiple covariates, the residual influence of BMI, lifestyles, and other potential confounding factors during pregnancy cannot be fully eliminated. Second, we did not measure the circulating levels of SCFAs and were not able to explore its role in the host‐microbiome interactions. Third, since the food questionnaire only covered major food groups, it prevented us from conducting further nutrient‐based (e.g., plant fiber) analysis. Finally, our participants were mostly residents of western China, which limited the extrapolation of our results. Though the cross‐validation strategy was used to avoid overfitting, the random forest models were not externally validated due to the lack of validation population in the research area of the gut metagenome and GDM. Further intervention studies or larger‐scale population‐based observational studies are needed to validate our findings.

## 3 Conclusion

Our study demonstrated a different gut microbiome remodeling pattern with advancing gestation in GDM patients compared with controls. Several microbial features, especially those related to dietary fiber fermentation, were identified to be associated with GDM status and host glucose metabolism. These findings may help understand the etiology of GDM from the perspectives of the interplay between the gut microbiome and host glycemic regulations.

## 4 Experimental Section

### Study Design and Population

This case‐control study was conducted within the TSBC, an ongoing prospective study conducted in the Shuangliu District of Chengdu, China, since 2017.^[^ ^46^ ^]^ The inclusion and exclusion criteria of participants was described previously.^[^ ^47^ ^]^ Briefly, pregnant women aged 18–40 years who attended initial prenatal care clinics during early pregnancy (≤15 weeks of gestation) at Shuangliu Maternal and Child Health Hospital were invited to participate. In each trimester of pregnancy (T1: <15+6, T2: 24+0–28+0, and T3: >29+0 weeks+days of gestation), enrolled participants attended the antenatal clinic, provided blood and fecal samples, completed a lifestyle questionnaire, and were interviewed by trained staff to record the dietary information for the recent three months using a food questionnaire of major food groups (i.e., grain, fruit, vegetables, meat, egg, and dairy product). GDM was diagnosed in T2 using 75‐g OGTT according to IADPSG criteria (blood glucose thresholds for GDM diagnosis: fasting 5.1 mmol/l, 1‐h 10.0 mmol/l, and 2‐h 8.5 mmol/l).^[^ ^48^ ^]^ By June 2019, 346 GDM patients were documented in the TSBC.

In the current analysis, women were excluded who: 1) had a prior diagnosis of T2DM; 2) used antibiotics or anti‐diabetic medication during pregnancy (e.g., Metformin and insulin); 3) failed to finish a complete food questionnaire; 4) failed to provide one fecal sample in each trimester. In total, 120 GDM patients were included and one pregnant woman was randomly matched who had normal glucose tolerance during pregnancy (1:1) with controlled age (±3 years), gestational weeks (±3 weeks), and date of baseline fecal sample collection (±4 weeks) (Figure 1). The basic characteristics and clinical measurements at baseline were not different between the included and excluded GDM cases (*P* > 0.05, Table S4, Supporting Information).

This study was approved by the Ethics Committee of Tongji Medical College, Huazhong University of Science and Technology, Wuhan, China, and carried out following the principles of the Declaration of Helsinki. Written informed consent was obtained from each participant before enrollment.

### Laboratory Measurements and Clinical Data Collection

Glycemic traits (i.e., FPG and HbA1c) and anthropometrics were measured in each trimester. Plasma glucose was measured using the Glucose Assay Kit (Sichuan Maccura Biotechnology, China) via the GOD‐PAP (glucose oxidase‐phenol and 4 aminophenazone) method. HbA1c was measured using a DCA Vantage Analyzer (Siemens Healthcare Diagnostics, Germany). The measurements of anthropometrics and covariates (i.e., BMI, blood pressure, physical activity, reproductive factors, and disease history) were described previously.^[^ ^47^ ^]^ GWG were calculated the difference between weight at each trimester and pre‐pregnancy weight.

### Fecal Sample Collection and Shotgun Metagenomic Profiling

Fecal samples were collected using sterile containers with ice boxes at the clinic and stored at −40 °C within 2 h after collection. Frozen fecal samples were transported with dry ice to the central laboratory and stored at −80 °C until processing. A total of 720 stool samples were collected for the following metagenomic sequencing in the current study. Details on fecal sample collection were described previously.^[^ ^11^ ^]^

Fecal DNA was extracted using the TIANamp Stool DNA kit (TIANGEN, Beijing, China) according to the experimental protocols. Illumina sequencing libraries (paired‐end, insert size: 350 bp) were prepared using the Tn5 DNA Library Prep Kit for Illumina (APExBIO, Boston, USA) according to the manufacturer's protocols.^[^ ^49^ ^]^ All libraries were sequenced on the Illumina Novaseq6000 platform (read length: 150 bp).

The quality control process of whole‐genome shotgun sequencing data was performed by KneadData (version 0.7.2), Trimmomatic (version 0.33), and Bowtie2 (version 2.3.4.3).^[^ ^50^, ^51^ ^]^ Human reads and rDNA reads were filtered by mapping the reads to the human reference genome (GRCh37) and SILVA 128 database. The trimmed nonhuman reads shorter than 75 bp were also removed. After quality control, an average of 33.4 million (min: 11.7 million, max: 51.0 million) high‐quality reads were obtained for each sample. The taxonomic profiles were determined by MetaPhlan (version 3.0.3),^[^ ^52^ ^]^ and the microbial functional profiles including MetaCyc pathways and Enzyme Commission gene families were determined by HUMAnN (version 3.0.0.alpha.3).^[^ ^53^ ^]^ Microbial species with a relative abundance < 0.01% in over 90% of all samples were excluded from the downstream analyses. The filtration of microbial pathways were described elsewhere.^[^ ^6^ ^]^ In brief, the pathways were excluded with a lower median abundance (< median abundance of all identified pathways) or with a relative abundance < 0.001% in over 90% of all samples, clustered the remaining pathways at the height of 0.6 using the R function “cutree”, and finally selected the representative pathways for each cluster (defined as the pathways with the median mean abundance). Eventually, a total of 258 microbial species and 138 pathways were included in the following analyses (Tables S2 and S3, Supporting Information).

To assess the overall association of gut microbial species with GDM, a composite microbial risk score for GDM was calculated based on the presence of 10 GDM‐related microbial species identified in the multivariate linear mixed model (for each of the 5 GDM‐depleted species [i.e., they were potential beneficial]: absent 0, present ‐1; for the GDM‐enriched species [i.e., they were potential harmful]: absent 0, present 1; ranged from −5 to 5).

### NMR‐Based Measurements of Major Fecal Short Chain Fatty Acids

Each fecal sample (about 50 mg) was extracted twice with 500 µL of phosphate buffer (0.15 m, K_2_HPO_4_/NaH_2_PO_4_ = 4:1, 0.001% w/v TSP, 0.01% w/v NaN_3_, 50% v/v D_2_O, pH 7.42), according to an optimized protocol.^[^ ^54^, ^55^, ^56^ ^]^ Then 500 µL of the final supernatant was transferred into a 5 mm NMR tube for NMR analysis, performed at 298 K on a Bruker Ascend^TM^ 600 MHz NMR spectrometer (600.13 MHz for ^1^H frequency) (Bruker Biospin, Germany). 1D ^1^H NMR spectra were acquired and processed as previously described using TopSpin 3.5 (Bruker Biospin, Germany) with ≈13 µs 90° pulses and 0.3 Hz exponential line broadening.^[^ ^54^ ^]^ Metabolites were assigned via 2D NMR spectra of pooled samples, with reference to literatures.^[^ ^57^, ^58^, ^59^ ^]^ For a target metabolite, one characteristic peak was chosen for curve fitting and integration using MestReNova (version 9.0.1, Mestrelab Research S. L., Spain),^[^ ^60^ ^]^ i.e., *δ* 0.90 (t) for butyrate, *δ* 1.92 (s) for acetate, and *δ* 2.19 (q) for propionate. The integral area of each metabolite was normalized by the number of protons corresponding to the peak and by the weight of feces used for extraction, which was utilized as the relative concentration for subsequent data analysis. The relative concentration of total SCFAs was calculated by summing up three major SCFAs, and the molar ratios of each SCFA were also calculated by dividing the relative concentration of single SCFA by that of total SCFAs.

### Statistical Analysis

The *α*‐diversity of gut microbiome was represented by Shannon index calculated using species‐level relative abundance matrix for each sample. The *β*‐diversity of gut microbiome between samples was calculated using species‐level Bray‐Curtis distance and was visualized using principal coordinate analysis (PCOA). The differences in microbial composition between different groups or trimesters were calculated using permutational multivariate analysis of variance (PERMANOVA) with a permutation of 9999 times via the R package “vegan” (version 2.5‐6). The variation of glycemic traits explained by microbial features and host factors was calculated using univariate linear regression. The first two eigenvalues of PCOA, which jointly explained more than 90% of the variation of the microbial community, were used to represent the *β*‐diversity of the gut microbiome in the above linear models.

A two‐step analysis was performed to explore the longitudinal associations between microbial features (i.e., microbial species and functional pathways) and host phenotypes (e.g., GDM status, FPG, HbA1c, BMI, and GWG) according to a framework described previously.^[^ ^61^ ^]^ First, based on a combination of data collected from three trimesters, the overall associations were estimated using MaAsLin2 (version 1.8.0) with linear mixed models (joint association). All models included each participant's identifier as the random effect and adjusted for potential covariates including maternal age, gestational age, pre‐pregnancy BMI, physical activity level, and smoking status as fixed effects. Second, the dynamic associations were estimated based on the temporal changes in microbial features from T1 to T2 and the OGTT results in T2 (i.e., glucose values at fasting, 1 h, and 2 h, and the glucose area under the curve [AUC]) with adjustment of maternal age and pre‐pregnancy BMI (delta association). Before association analyses, the relative abundance of microbial species and functional pathways was transformed using the arc‐sin square root transformation,the fecal levels of SCFAs were transformed using inverse normal transformation, and the host phenotype data were scaled into Z‐scores. All *P* values for multiple hypothesis testing were adjusted for multiple comparisons using the Benjamini‐Hochberg method, and a FDR‐corrected *P* < 0.25 was considered statistically significant.

Given the physiological links among diet, fecal microbiome, and host metabolism, interaction effects between diet and fecal microbiota were estimated by adding an interaction term into the regression model. The term consisted of dietary levels (categorical: lower or higher classified by median intake amount of each food group) and microbial features (continuous: relative abundance). The linear mixed models and covariates used in the interaction analysis were the same as those used in the aforementioned joint association analysis. A *P* < 0.05 of the interaction term was regarded as statistically significant.

To explore the value of differential microbial features in T1 in predicting the risk of GDM, we constructed random forest classifiers for GDM diagnosis and random forest regression models for glycemic traits measured in T2. We also assessed the ability of the microbial parity features to increase the power in predicting GDM in addition to the traditional risk factors in T1 (i.e., maternal age, BMI, physical activity levels, FPG, HbA1c, blood pressure, history of polycystic ovary syndrome, family history of T2DM, and parity).^[^ ^62^ ^]^ The random forest models were constructed using a 5‐fold cross‐validation algorithm. The parameters of random forest models were tuned using the R package “caret”. The AUROC was used as a metric to quantify classifiers’ performance. The significance of the comparison between model performances was assessed using the Delong test via the “roc.test” function of the R package “pROC” (version 1.0‐11). The correlation between the predicted value and the observed value was used to estimate the performance of the regression model. All data analyses were conducted in R (version 4.1.1).

## Conflict of Interest

The authors declare no conflict of interest.

## Author Contributions

Z.S. and X.‐F.P. contributed equally to this work. A.P. and Y.Z. contributed to the design and interpretation of the study. Z.H.S and X.‐F.P. contributed to the formal analysis and drafted the first version of the manuscript. X.‐F.P., Y.W., Y.Y., P.W., B.Z., and J.G.X. contributed to data collection. Z.H.S., X.L., M.M.K., Y.N.P., M.Y.Z., and J.Y.H. contributed to data curation. A.P., Y.Z., Z.H.S., X.‐F.P., L.M.J., P.H., J.F.W., G.C.C., C.Z.Y., Y.F.Y., X.G., and F.Q.Z. provided critically important revisions to the manuscripts. All authors revised the manuscripts, approved the final version of the manuscript, and the submission of the manuscript. Y.Z. is the guarantor of this work and, as such, had full access to all the data in the study and takes responsibility for the integrity of the data and the accuracy of the data analysis.

## Supporting information

Supporting Information

## Data Availability

The metagenomics data that support the findings of this study are available in NODE (http://www.biosino.org/node, Project ID: OEP003504).

## References

[1] H. D. McIntyre , P. Catalano , C. Zhang , G. Desoye , E. R. Mathiesen , P. Damm , Nat. Rev. Dis. Primers 2019, 5, 47. 31296866 10.1038/s41572-019-0098-8

[2] K. K. Venkatesh , C. D. Lynch , C. E. Powe , M. M. Costantine , S. F. Thung , S. G. Gabbe , W. A. Grobman , M. B. Landon , JAMA, J. Am. Med. Assoc. 2022, 327, 1356. 10.1001/jama.2022.3189 PMC9006108 35412565

[3] E. Vounzoulaki , K. Khunti , S. C. Abner , B. K. Tan , M. J. Davies , C. L. Gillies , BMJ 2020, 369, m1361. 32404325 10.1136/bmj.m1361 PMC7218708

[4] T. D. Clausen , E. R. Mathiesen , T. Hansen , O. Pedersen , D. M. Jensen , J. Lauenborg , P. Damm , Diabetes Care 2008, 31, 340. 18000174 10.2337/dc07-1596

[5] W. Ye , C. Luo , J. Huang , C. Li , Z. Liu , F. Liu , BMJ 2022, 377, e067946. 35613728 10.1136/bmj-2021-067946 PMC9131781

[6] L. B. Thingholm , M. C. Ruhlemann , M. Koch , B. Fuqua , G. Laucke , R. Boehm , C. Bang , E. A. Franzosa , M. Hubenthal , A. Rahnavard , F. Frost , J. Lloyd‐Price , M. Schirmer , A. J. Lusis , C. D. Vulpe , M. M. Lerch , G. Homuth , T. Kacprowski , C. O. Schmidt , U. Nothlings , T. H. Karlsen , W. Lieb , M. Laudes , A. Franke , C. Huttenhower , Cell Host Microbe 2019, 26, 252. 31399369 10.1016/j.chom.2019.07.004 PMC7720933

[7] M. C. Collado , E. Isolauri , K. Laitinen , S. Salminen , Am. J. Clin. Nutr. 2008, 88, 894. 18842773 10.1093/ajcn/88.4.894

[8] X. Wang , H. Liu , Y. Li , S. Huang , L. Zhang , C. Cao , P. N. Baker , C. Tong , P. Zheng , H. Qi , Gut Microbes 2020, 12, 1840765. 33222612 10.1080/19490976.2020.1840765 PMC7714515

[9] W. Zheng , Q. Xu , W. Huang , Q. Yan , Y. Chen , L. Zhang , Z. Tian , T. Liu , X. Yuan , C. Liu , J. Luo , C. Guo , W. Song , L. Zhang , X. Liang , H. Qin , G. Li , mSystems 2020, 5, e00109. 32209715 10.1128/mSystems.00109-20 PMC7093821

[10] K. Mokkala , N. Paulin , N. Houttu , E. Koivuniemi , O. Pellonpera , S. Khan , S. Pietila , K. Tertti , L. L. Elo , K. Laitinen , Gut 2021, 70, 309. 32839200 10.1136/gutjnl-2020-321643

[11] P. Hu , X. Chen , X. Chu , M. Fan , Y. Ye , Y. Wang , M. Han , X. Yang , J. Yuan , L. Zha , B. Zhao , C.‐X. Yang , X.‐R. Qi , K. Ning , J. Debelius , W. Ye , B. Xiong , X.‐F. Pan , A. Pan , J. Clin. Endocrinol. Metab. 2021, 106, e4128. 34015117 10.1210/clinem/dgab346

[12] S. Yan , R. Shi , L. Li , S. Ma , H. Zhang , J. Ye , J. Wang , J. Pan , Q. Wang , X. Jin , X. Liu , Z. Liu , Mol. Nutr. Food Res. 2019, 63, 1900521. 10.1002/mnfr.201900521 31487425

[13] D. D. Wang , L. H. Nguyen , Y. Li , Y. Yan , W. Ma , E. Rinott , K. L. Ivey , I. Shai , W. C. Willett , F. B. Hu , E. B. Rimm , M. J. Stampfer , A. T. Chan , C. Huttenhower , Nat. Med. 2021, 27, 333. 33574608 10.1038/s41591-020-01223-3 PMC8186452

[14] D. A. Alarcon , M. Nandi , X. Carpena , I. Fita , P. C. Loewen , Acta Crystallogr. Sect. F Struct. Biol. Cryst. Commun. 2012, 68, 1279. 10.1107/S1744309112037736 PMC3515364 23143232

[15] S. Wulfert , S. Schilasky , S. Krueger , Plants 2020, 9, 353. 32168758 10.3390/plants9030353 PMC7154858

[16] C. Wang , Y. Xiao , L. Yu , F. Tian , J. Zhao , H. Zhang , W. Chen , Q. Zhai , J. Adv. Res. 2022, 36, 27. 35127162 10.1016/j.jare.2021.06.012 PMC8799915

[17] G. Mao , S. Li , C. Orfila , X. Shen , S. Zhou , R. J. Linhardt , X. Ye , S. Chen , Food Funct. 2019, 10, 7828. 31778135 10.1039/c9fo01534e

[18] M. K. W. Crusell , T. H. Hansen , T. Nielsen , K. H. Allin , M. C. Ruhlemann , P. Damm , H. Vestergaard , C. Rorbye , N. R. Jorgensen , O. B. Christiansen , F.‐A. Heinsen , A. Franke , T. Hansen , J. Lauenborg , O. Pedersen , Microbiome 2018, 6, 89. 29764499 10.1186/s40168-018-0472-x PMC5952429

[19] J. Wang , J. Zheng , W. Shi , N. Du , X. Xu , Y. Zhang , P. Ji , F. Zhang , Z. Jia , Y. Wang , Z. Zheng , H. Zhang , F. Zhao , Gut 2018, 67, 1614. 29760169 10.1136/gutjnl-2018-315988 PMC6109274

[20] M. Tang , N. E. Weaver , D. N. Frank , D. Ir , C. E. Robertson , J. F. Kemp , J. Westcott , K. Shankar , A. L. Garces , L. Figueroa , A. K. Tshefu , A. L. Lokangaka , S. S. Goudar , M. Somannavar , S. Aziz , S. Saleem , E. M. McClure , K. M. Hambidge , A. E. Hendricks , N. F. Krebs , Front. Microbiol. 2022, 13, 823757. 35979501 10.3389/fmicb.2022.823757 PMC9376441

[21] C. B. Miller , P. Benny , J. Riel , C. Boushey , R. Perez , V. Khadka , Y. Qin , A. K. Maunakea , M.‐J. Lee , BMC Pregnancy Childbirth 2021, 21, 558. 34399704 10.1186/s12884-021-04033-8 PMC8369757

[22] O. Koren , J. K. Goodrich , T. C. Cullender , A. Spor , K. Laitinen , H. K. Backhed , A. Gonzalez , J. J. Werner , L. T. Angenent , R. Knight , F. Backhed , E. Isolauri , S. Salminen , R. E. Ley , Cell 2012, 150, 470. 22863002 10.1016/j.cell.2012.07.008 PMC3505857

[23] J.‐H. Shin , Y.‐H. Park , M. Sim , S.‐A. Kim , H. Joung , D.‐M. Shin , Res. Microbiol. 2019, 170, 192. 30940469 10.1016/j.resmic.2019.03.003

[24] S. Stojanov , A. Berlec , B. Strukelj , Microorganisms 2020, 8, 1715. 33139627 10.3390/microorganisms8111715 PMC7692443

[25] K. Y. Sugino , T. L. Hernandez , L. A. Barbour , J. M. Kofonow , D. N. Frank , J. E. Friedman , Front. Endocrinol. 2022, 13, 921464. 10.3389/fendo.2022.921464 PMC9366142 35966074

[26] C. Festa , L. Drago , M. Martorelli , V. P. Di Marino , O. Bitterman , C. C. Corleto , V. D. Corleto , A. Napoli , New Microbiol. 2020, 43, 195. 33135080

[27] S. Priya , M. B. Burns , T. Ward , R. A. T. Mars , B. Adamowicz , E. F. Lock , P. C. Kashyap , D. Knights , R. Blekhman , Nat. Microbiol. 2022, 7, 780. 35577971 10.1038/s41564-022-01121-z PMC9159953

[28] B. J. Parker , P. A. Wearsch , A. C. M. Veloo , A. Rodriguez‐Palacios , Front. Immunol. 2020, 11, 906. 32582143 10.3389/fimmu.2020.00906 PMC7296073

[29] Y. Wu , P. W. Bible , S. Long , W.‐k. Ming , W. Ding , Y. Long , X. Wen , X. Li , X. Deng , Y. Deng , S. Guo , C. L. Doci , L. Wei , H. Chen , Z. Wang , Acta Diabetol. 2020, 57, 569. 31820107 10.1007/s00592-019-01434-2

[30] D. Zhao , H. Zhu , F. Gao , Z. Qian , W. Mao , Y. Yin , J. Tan , D. Chen , Food Funct. 2020, 11, 6528. 32638790 10.1039/d0fo00180e

[31] J. Wei , Y. Qing , H. Zhou , J. Liu , C. Qi , J. Gao , J. Endocrinol. Invest. 2021, 45, 279. 34302684 10.1007/s40618-021-01595-4 PMC8308075

[32] X. Ze , S. H. Duncan , P. Louis , H. J. Flint , ISME J 2012, 6, 1535. 22343308 10.1038/ismej.2012.4 PMC3400402

[33] M. D. Robertson , A. S. Bickerton , A. L. Dennis , H. Vidal , K. N. Frayn , Am. J. Clin. Nutr. 2005, 82, 559. 16155268 10.1093/ajcn.82.3.559

[34] K. E. Bach Knudsen , H. N. Laerke , M. S. Hedemann , T. S. Nielsen , A. K. Ingerslev , D. S. Gundelund Nielsen , P. K. Theil , S. Purup , S. Hald , A. G. Schioldan , M. L. Marco , S. Gregersen , K. Hermansen , Nutrients 2018, 10, 1499. 30322146 10.3390/nu10101499 PMC6213552

[35] M. Rautio , E. Eerola , M.‐L. Vaisanen‐Tunkelrott , D. Molitoris , P. Lawson , M. D. Collins , H. Jousimies‐Somer , Syst. Appl. Microbiol. 2003, 26, 182. 12866844 10.1078/072320203322346029

[36] V. K. Ridaura , J. J. Faith , F. E. Rey , J. Cheng , A. E. Duncan , A. L. Kau , N. W. Griffin , V. Lombard , B. Henrissat , J. R. Bain , M. J. Muehlbauer , O. Ilkayeva , C. F. Semenkovich , K. Funai , D. K. Hayashi , B. J. Lyle , M. C. Martini , L. K. Ursell , J. C. Clemente , W. Van Treuren , W. A. Walters , R. Knight , C. B. Newgard , A. C. Heath , J. I. Gordon , Science 2013, 341, 1241214. 24009397 10.1126/science.1241214 PMC3829625

[37] M. O. Ruuskanen , P. P. Erawijantari , A. S. Havulinna , Y. Liu , G. Meric , J. Tuomilehto , M. Inouye , P. Jousilahti , V. Salomaa , M. Jain , R. Knight , L. Lahti , T. J. Niiranen , Diabetes Care 2022, 45, 811. 35100347 10.2337/dc21-2358 PMC9016732

[38] A. Metwaly , S. Reitmeier , D. Haller , Nat. Rev. Gastroenterol. Hepatol. 2022, 19, 383. 35190727 10.1038/s41575-022-00581-2

[39] Y. Zhang , Y. Gu , H. Ren , S. Wang , H. Zhong , X. Zhao , J. Ma , X. Gu , Y. Xue , S. Huang , J. Yang , L. Chen , G. Chen , S. Qu , J. Liang , L. Qin , Q. Huang , Y. Peng , Q. Li , X. Wang , P. Kong , G. Hou , M. Gao , Z. Shi , X. Li , Y. Qiu , Y. Zou , H. Yang , J. Wang , G. Xu , et al., Nat. Commun. 2020, 11, 5015. 33024120 10.1038/s41467-020-18414-8 PMC7538905

[40] L. Zhao , F. Zhang , X. Ding , G. Wu , Y. Y. Lam , X. Wang , H. Fu , X. Xue , C. Lu , J. Ma , L. Yu , C. Xu , Z. Ren , Y. Xu , S. Xu , H. Shen , X. Zhu , Y. Shi , Q. Shen , W. Dong , R. Liu , Y. Ling , Y. Zeng , X. Wang , Q. Zhang , J. Wang , L. Wang , Y. Wu , B. Zeng , H. Wei , et al., Science 2018, 359, 1151. 29590046 10.1126/science.aao5774

[41] L. R. S. Moreira , E. X. F. Filho , Appl. Microbiol. Biotechnol. 2008, 79, 165. 18385995 10.1007/s00253-008-1423-4

[42] X. Han , Nat. Rev. Endocrinol. 2016, 12, 668. 27469345 10.1038/nrendo.2016.98

[43] E. E. Canfora , J. W. Jocken , E. E. Blaak , Nat. Rev. Endocrinol. 2015, 11, 577. 26260141 10.1038/nrendo.2015.128

[44] A. Koh , F. De Vadder , P. Kovatcheva‐Datchary , F. Backhed , Cell 2016, 165, 1332. 27259147 10.1016/j.cell.2016.05.041

[45] J. Lloyd‐Price , C. Arze , A. N. Ananthakrishnan , M. Schirmer , J. Avila‐Pacheco , T. W. Poon , E. Andrews , N. J. Ajami , K. S. Bonham , C. J. Brislawn , D. Casero , H. Courtney , A. Gonzalez , T. G. Graeber , A. B. Hall , K. Lake , C. J. Landers , H. Mallick , D. R. Plichta , M. Prasad , G. Rahnavard , J. Sauk , D. Shungin , Y. Vazquez‐Baeza , R. A. 3rd White , I. Investigators , J. Braun , L. A. Denson , J. K. Jansson , R. Knight , et al., Nature 2019, 569, 655. 31142855 10.1038/s41586-019-1237-9 PMC6650278

[46] Y. Wang , Y. Huang , P. Wu , Y. Ye , F. Sun , X. Yang , Q. Lu , J. Yuan , Y. Liu , H. Zeng , X. Song , S. Yan , X. Qi , C.‐X. Yang , C. Lv , J. H. Y. Wu , G. Liu , X.‐F. Pan , D. Chen , A. Pan , Am. J. Clin. Nutr. 2021, 114, 1763. 34477820 10.1093/ajcn/nqab242

[47] X.‐F. Pan , Y. Huang , X. Li , Y. Wang , Y. Ye , H. Chen , M. Marklund , Y. Wen , Y. Liu , H. Zeng , X. Qi , X. Yang , C.‐X. Yang , G. Liu , R. A. Gibson , S. Xu , D. Yu , D. Chen , Y. Li , Z. Mei , A. Pan , J. H. Y. Wu , Eur. J. Endocrinol. 2021, 185, 87. 33914701 10.1530/EJE-21-0118

[48] D. International Association of , P. Pregnancy Study Groups Consensus Panel , B. E. Metzger , Diabetes Care 2010, 33, 676. 20190296 10.2337/dc09-1848 PMC2827530

[49] J. Qin , Y. Li , Z. Cai , S. Li , J. Zhu , F. Zhang , S. Liang , W. Zhang , Y. Guan , D. Shen , Y. Peng , D. Zhang , Z. Jie , W. Wu , Y. Qin , W. Xue , J. Li , L. Han , D. Lu , P. Wu , Y. Dai , X. Sun , Z. Li , A. Tang , S. Zhong , X. Li , W. Chen , R. Xu , M. Wang , Q. Feng , et al., Nature 2012, 490, 55. 23023125 10.1038/nature11450

[50] B. Langmead , S. L. Salzberg , Nat. Methods 2012, 9, 357. 22388286 10.1038/nmeth.1923 PMC3322381

[51] A. M. Bolger , M. Lohse , B. Usadel , Bioinformatics 2014, 30, 2114. 24695404 10.1093/bioinformatics/btu170 PMC4103590

[52] N. Segata , L. Waldron , A. Ballarini , V. Narasimhan , O. Jousson , C. Huttenhower , Nat. Methods 2012, 9, 811. 22688413 10.1038/nmeth.2066 PMC3443552

[53] R. Caspi , R. Billington , C. A. Fulcher , I. M. Keseler , A. Kothari , M. Krummenacker , M. Latendresse , P. E. Midford , Q. Ong , W. K. Ong , S. Paley , P. Subhraveti , P. D. Karp , Nucleic Acids Res. 2017, 46, D633. 10.1093/nar/gkx935 PMC5753197 29059334

[54] L. Jiang , J. Wang , R. Li , Z.‐m. Fang , X.‐H. Zhu , X. Yi , H. Lan , X. Wei , D.‐S. Jiang , Metabolomics 2019, 15, 57. 30937548 10.1007/s11306-019-1518-1

[55] Y. Zhao , J. Wu , J. V. Li , N.‐Y. Zhou , H. Tang , Y. Wang , J. Proteome Res. 2013, 12, 2987. 23631562 10.1021/pr400263n

[56] J. Wu , Y. An , J. Yao , Y. Wang , H. Tang , Analyst 2010, 135, 1023. 20419252 10.1039/b927543f

[57] L. Jiang , R. Ramamoorthy , S. Ramachandran , P. P. Kumar , Int. J. Mol. Sci. 2020, 21, 1924. 32168953 10.3390/ijms21061924 PMC7139402

[58] J. V. Li , H. Ashrafian , M. Bueter , J. Kinross , C. Sands , C. W. le Roux , S. R. Bloom , A. Darzi , T. Athanasiou , J. R. Marchesi , J. K. Nicholson , E. Holmes , Gut 2011, 60, 1214. 21572120 10.1136/gut.2010.234708 PMC3677150

[59] M. Cui , A. Trimigno , V. Aru , B. Khakimov , S. B. Engelsen , Anal. Chem. 2020, 92, 9546. 32567838 10.1021/acs.analchem.0c00606

[60] L. Jiang , S. C. Lee , T. C. Ng , J. Proteome Res. 2018, 17, 1248. 29385795 10.1021/acs.jproteome.7b00859

[61] L. Chen , D. Wang , S. Garmaeva , A. Kurilshikov , A. Vich Vila , R. Gacesa , T. Sinha , Lifelines Cohort Study , E. Segal , R. K. Weersma , C. Wijmenga , A. Zhernakova , J. Fu , Cell 2021, 184, 2302. 33838112 10.1016/j.cell.2021.03.024

[62] M. Savvidou , S. M. Nelson , M. Makgoba , C.‐M. Messow , N. Sattar , K. Nicolaides , Diabetes 2010, 59, 3017. 20876721 10.2337/db10-0688 PMC2992761

