# Supplementary material for: The Gut Microbiome Dynamically Associates with Host Glucose Metabolism throughout Pregnancy: Longitudinal Findings from a Matched Case‐Control Study of Gestational Diabetes Mellitus
Source: Adv Sci (Weinh). 2023 Jan 22;10(10):2205289. doi: 10.1002/advs.202205289 (PMC10074094; doi:10.1002/advs.202205289)
Supplement: Supplementary file 1 — Supporting Information [file ADVS-10-2205289-s001.pdf]

Supporting Information

The Gut Microbiome Dynamically Associates with Host Glucose Metabolism throughout Pregnancy: Longitudinal Findings from a Matched Case-control Study of Gestational Diabetes Mellitus

Zhonghan Sun, Xiong-Fei Pan, Xiao Li, Limiao Jiang, Ping Hu, Yi Wang, Yi Ye, Ping Wu, Bin Zhao, Jianguo Xu, Mengmeng Kong, Yanni Pu, Manying Zhao, Jianying Hu, Jinfeng Wang, Guo-Chong Chen, Changzheng Yuan, Yongfu Yu, Xiang Gao, Fangqing Zhao, An Pan, Yan Zheng

Figure S1. The relative abundances of microbial of phylum among GDM cases and controls at each trimester of pregnancy.

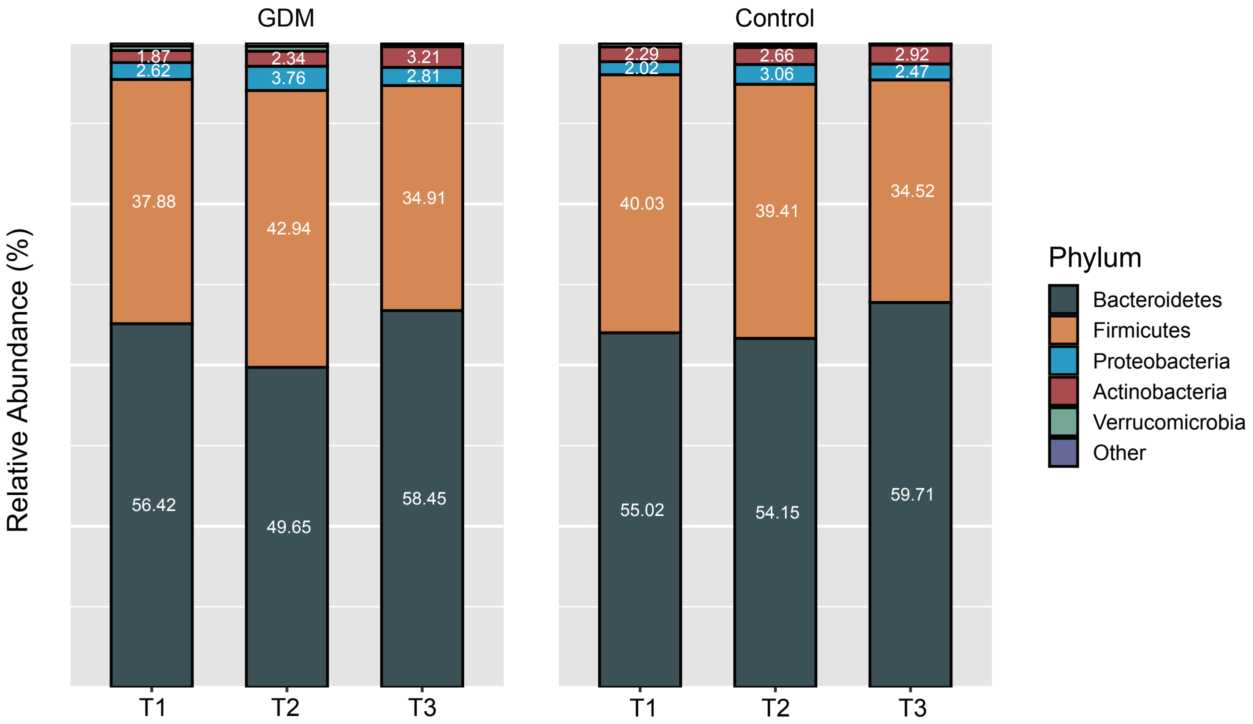

Figure S2. The composition of gut microbiome among GDM cases and control at each trimester. The α-diversity was represented by the Shannon index (A) and the β-diversity distance (B) was calculated using the Bray-Curtis distance, both of which were calculated with microbial data at the species level.

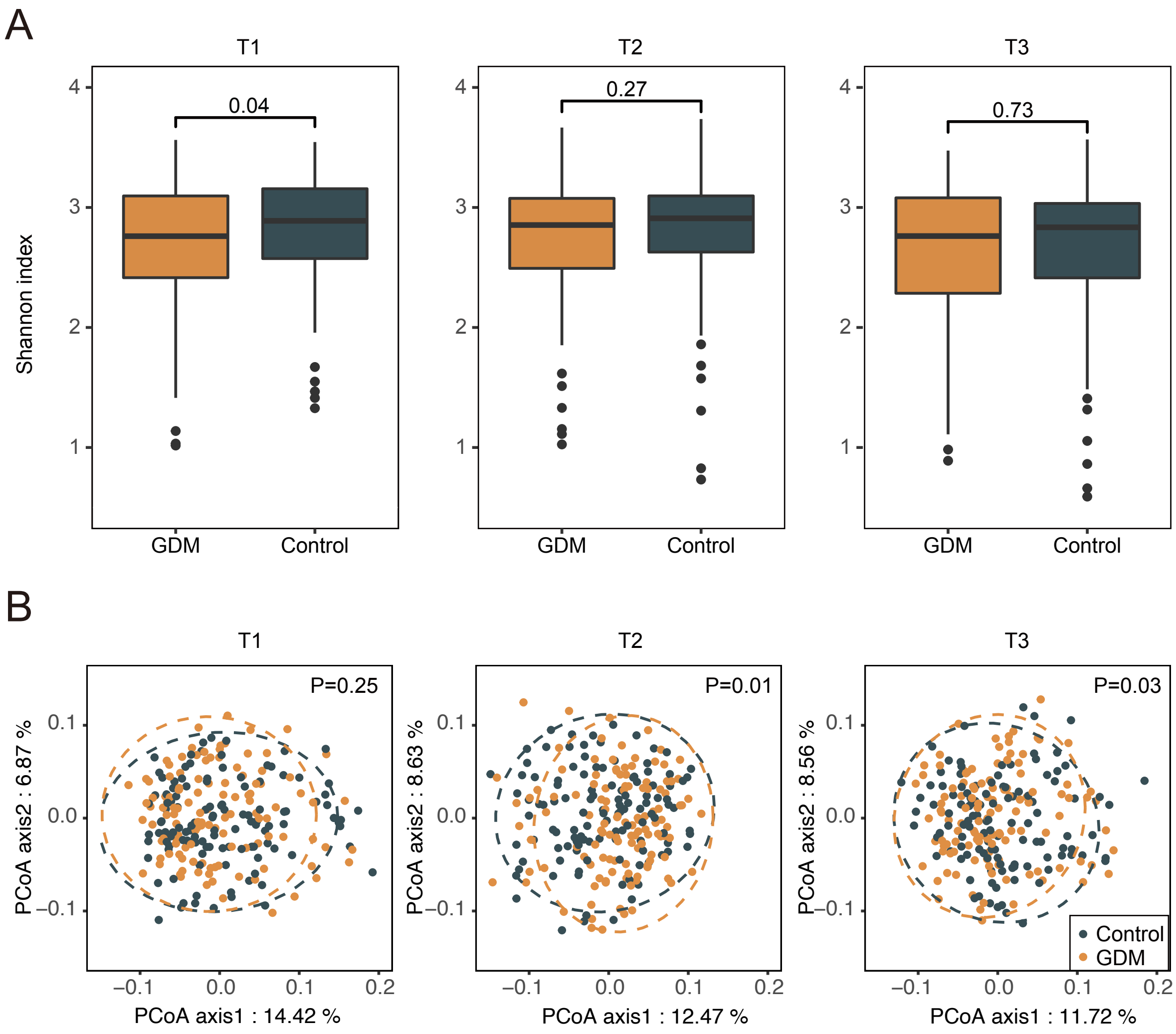

Figure S3. The distributions of GDM-related species in GDM patients and controls during pregnancy. (A) The microbial species differentially enriched between GDM patients and NGT controls at each trimester. Yellow bubbles represent the group in which the species had higher relative abundances. The bubble size represents the log- transformed fold change of the relative abundance between two groups (GDM vs. Control). (B) The temporal change of the relative abundances of GDM- related species in GDM cases and control during pregnancy.

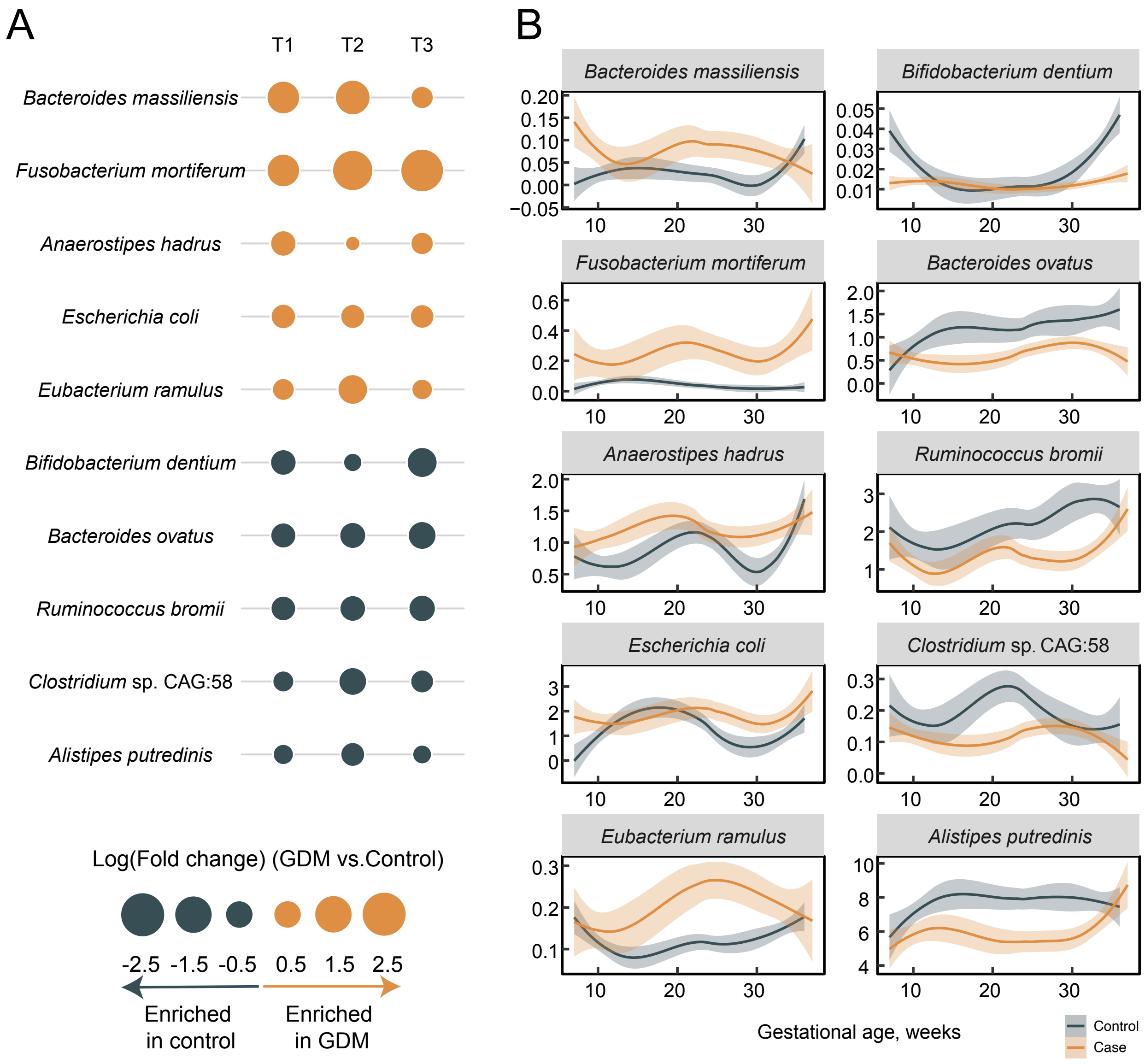

Figure S4. GDM-related microbial pathways associated with glucose metabolism. (A) The associations between GDM- related pathways and host glucose metabolism and BMI. Asterisks represent FDR < 0.25. (B) The microbial pathways differentially enriched between GDM patients and controls at each trimester. Yellow bubbles represent GDM- increased pathways and blue bubbles represent GDM-decreased pathways. The bubble size represents the log- transformed fold change of the relative abundance between the two groups. (C) The temporal change of the relative abundances of GDM-related species in GDM cases and control during pregnancy.

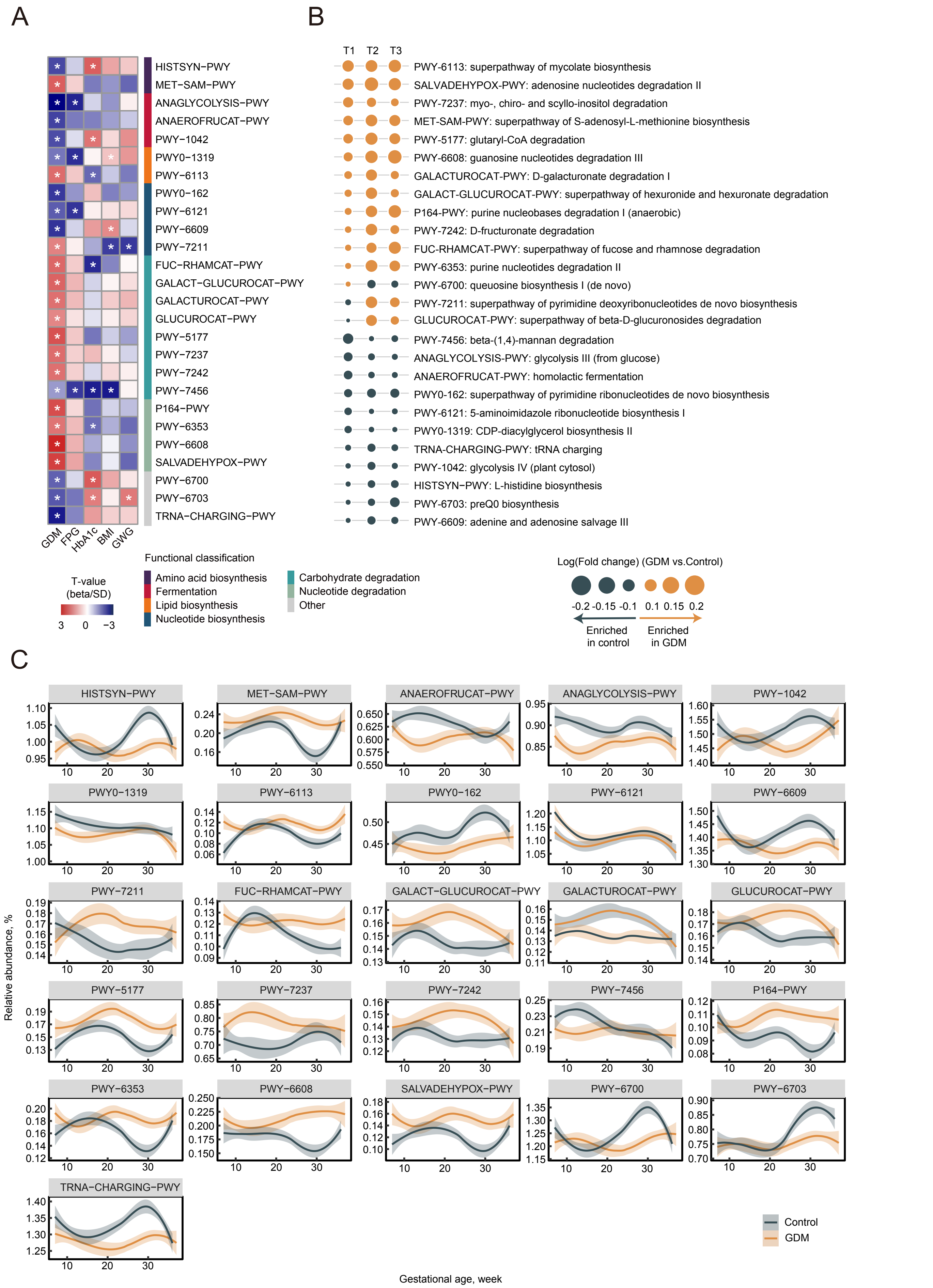

Figure S5. Fecal levels of major SCFAs during pregnancy. The left panels showed the SCFAs levels measured at each trimester, and the right panels showed the temporal change of SCFAs levels between the first and second trimesters.

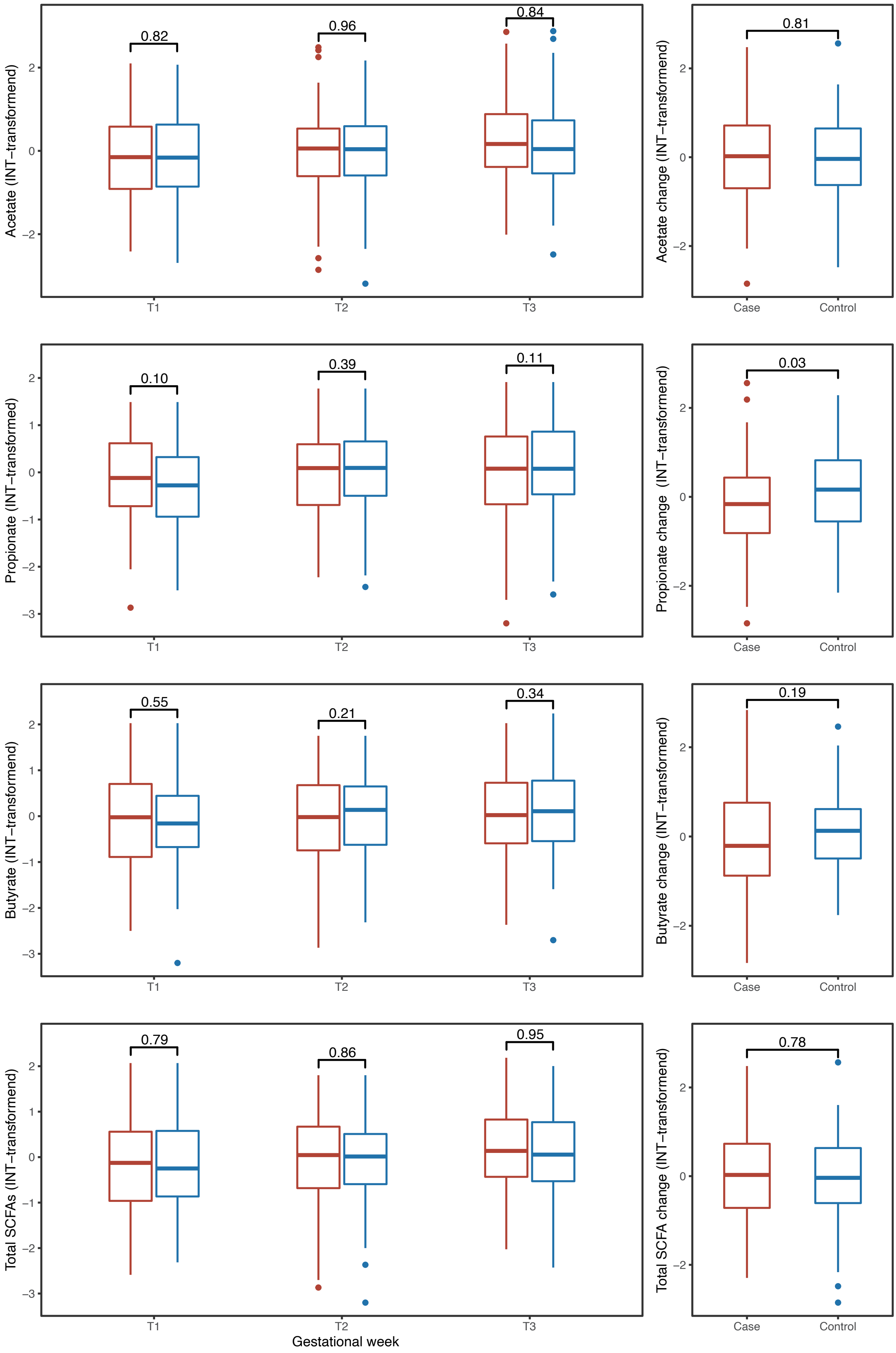

Figure S6. The associations between fecal levels of SCFAs and glucose metabolism. Asterisks represented *P* <0.25.

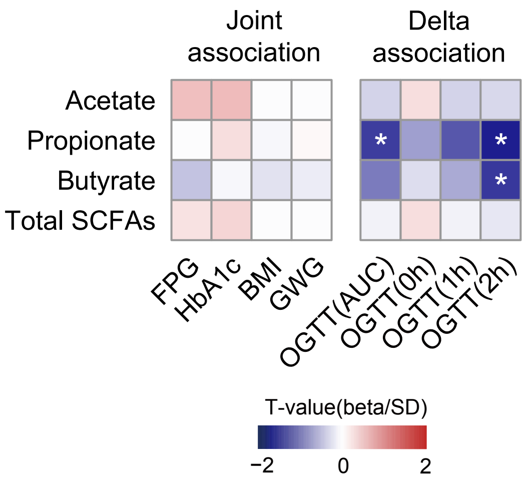

Table S1. Basic characteristics, clinical measurements and dietary intake amounts among GDM cases and controls at baseline and during the pregnancy.

	GDM n=120	Non-GDM n=120	*P*-value
**Age, years**	27.5 ± 4.0	27.3 ± 3.8	0.78
**Gestational age, weeks**			
First trimester	10.2 ± 2.0	10.4 ± 1.8	0.3
Second trimester	23.9 ± 1.6	24.0 ± 1.5	0.69
Third trimester	33.6 ± 1.2	33.7 ± 1.1	0.1
**Smoking history**			1
Current/Former	5 (4.2)	6 (4.9)	
Never	115 (95.8)	115 (95.1)	
**Drinking history**			0.25
Current/Former	22 (19.2)	15 (13.1)	
Never	98 (80.8)	105 (86.9)	
**Education**			0.74
Junior secondary	40 (33.3)	38 (31.1)	
Senior secondary	62 (51.7)	60 (50.0)	
Tertiary	18 (15.0)	22 (18.9)	
**Family history of type 2 diabetes**	6 (5.0)	7 (5.7)	1
**Parity**			1
0	61 (50.8)	58 (48.3)	
>1	59 (49.2)	62 (51.7)	
**BMI, kg/m^2**			
Prepregnancy	21.6 ± 3.1	20.8 ± 2.4	**0.039**
First trimester	22.0 ± 3.0	21.1 ± 2.5	**0.012**
Second trimester	24.2 ± 3.1	23.5 ± 2.5	**0.041**
Third trimester	25.7 ± 3.0	25.7 ± 2.4	0.89
**Gestational weight gain, kg**			
First trimester	1.2 ± 2.8	0.7 ± 2.2	0.18
Second trimester	6.6 ± 4.0	6.6 ± 3.0	0.96
Third trimester	10.4 ± 4.7	12.2 ± 3.3	**< 0.001**
**Physical activity levels, METs**			
First trimester	116.1 ± 65.0	131.4 ± 66.0	**0.04**
Second trimester	128.3 ± 78.3	128.3 ± 68.4	0.99
Third trimester	132.0 ± 64.4	135.5 ± 71.6	0.68
**Systolic blood pressure, mmHg**			
First trimester	111.6 ± 10.2	108.6 ± 8.6	**0.01**
Second trimester	112.4 ± 9.7	109.1 ± 8.8	**0.005**
Third trimester	110.8 ± 9.0	110.3 ± 9.0	0.62
**Diastolic blood pressure, mmHg**			
First trimester	74.5 ± 8.5	73.6 ± 7.4	0.24
Second trimester	72.4 ± 7.1	70.4 ± 6.7	**0.02**
Third trimester	75.2 ± 8.1	74.3 ± 7.3	0.51
**Fasting plasma glucose, mmol/L**			
First trimester	4.5 ± 0.5	4.3 ± 0.4	**0.002**
Second trimester	4.3 ± 0.6	3.9 ± 0.3	**< 0.001**

Third trimester	4.2 ± 0.5	4.3 ± 0.4	0.64
**OGTT 1h glucose, mmol/L**	10.0 ± 1.7	6.8 ± 1.5	< 0.001
**OGTT 2h glucose, mmol/L**	8.7 ± 1.7	5.9 ± 1.0	< 0.001
**Hemoglobin A1c, %**			
First trimester	5.2 ± 0.3	5.1 ± 0.2	**< 0.001**
Second trimester	4.8 ± 0.3	4.6 ± 0.3	**< 0.001**
Third trimester	5.1 ± 0.4	5.0 ± 0.3	**0.014**
**Refined Grain, serving/day**			
T1	3.3 ± 1.7	3.6 ± 1.8	0.13
T2	3.7 ± 1.7	3.9 ± 1.7	0.27
T3	2.3 ± 1.4	3.3 ± 1.5	**< 0.001**
**Vegetable, serving/day**			
T1	3.4 ±2.1	3.3 ±2.2	0.94
T2	4.0 ±1.9	3.6 ±2.1	0.08
T3	2.6 ±2.1	2.3 ±1.9	0.27
**Fruit, serving/day**			
T1	5.2 ±2.7	5.6 ±2.7	0.27
T2	5.4 ±2.7	5.3 ±2.7	0.87
T3	2.9 ±1.9	3.6 ±2.0	**0.003**
**Meat, serving/day**			
T1	0.9 ±0.6	1.1 ±0.7	0.17
T2	1.2 ±0.6	1.2 ±0.7	0.72
T3	1.4 ±0.9	1.3 ±0.6	0.27
**Egg, serving/day**			
T1	0.8 ±0.6	0.8 ±0.5	0.94
T2	0.9 ±0.5	0.9 ±0.6	0.94
T3	1.1 ±0.4	1.0 ±0.4	**0.03**
**Dairy product, serving/day**			
T1	192.4 ±125.4	200.6 ±111.0	0.95
T2	244.6 ±105.8	211.2 ±97.0	**0.02**
T3	295.3 ±164.3	264.2 ±133.1	**0.03**

Data were shown as Mean ± SD for continuous variables or N (%) for categorical variables. The group comparisons were conducted using Student's t-test or Wilcox ranked sum test for continuous variables and $\chi 2$ test or Fisher's exact test for categorical variables. Each serving for refined grain, fruit, vegetable and meat were defined as 50 gram, for egg as one count, and for diary product as 50 mililiter.

Table S2. The relative abundance and prevalence of microbial species included in the current analysis.

Species	T1					T2					T3				
	Mean case	Mean control	Prevalence	Wilcoxon P	Wilcoxon FDR	Mean case	Mean control	Prevalence	Wilcoxon P	Wilcoxon FDR	Mean case	Mean control	Prevalence	Wilcoxon P	Wilcoxon FDR
Ruminococcus bicirculans	4.1E-03	4.8E-03	0.63	0.00	0.09	3.4E-03	5.2E-03	0.61	0.17	0.36	6.4E-03	5.5E-03	0.60	0.81	0.49
Intestinibacter bartlettii	2.3E-04	2.2E-04	0.28	0.00	0.17	3.8E-04	2.9E-04	0.36	0.73	0.49	1.3E-04	2.6E-04	0.27	0.18	0.37
Methanobrevibacter smithii	7.6E-04	3.4E-05	0.11	0.00	0.21	3.9E-03	9.6E-04	0.10	0.10	0.35	4.2E-04	5.6E-04	0.11	0.64	0.48
Ruminococcus bromii	1.1E-02	1.6E-02	0.73	0.00	0.24	1.4E-02	2.3E-02	0.81	0.01	0.10	1.7E-02	2.9E-02	0.80	0.01	0.15
Adlercreutzia equolifaciens	1.2E-03	1.1E-03	0.71	0.01	0.24	1.2E-03	1.4E-03	0.67	0.90	0.49	7.8E-04	7.7E-04	0.62	0.46	0.43
Muribaculum intestinale	1.3E-04	1.1E-04	0.31	0.01	0.24	9.4E-05	9.6E-05	0.28	0.20	0.36	1.1E-04	1.2E-04	0.30	0.40	0.42
Gordonia jacobaea	1.1E-04	7.9E-05	0.25	0.01	0.24	6.4E-05	5.7E-05	0.19	0.09	0.35	8.3E-05	6.2E-05	0.21	0.29	0.40
Bacteroides plebeius	2.8E-02	4.0E-02	0.75	0.01	0.24	2.1E-02	3.3E-02	0.73	0.49	0.49	2.3E-02	3.8E-02	0.72	0.19	0.37
Bifidobacterium longum	7.3E-04	2.2E-03	0.38	0.01	0.24	1.5E-03	2.4E-03	0.38	0.04	0.25	9.2E-04	1.7E-03	0.39	0.19	0.37
Firmicutes bacterium CAG 95	1.5E-03	1.3E-03	0.36	0.01	0.24	4.2E-04	1.0E-03	0.31	0.09	0.35	1.4E-03	9.7E-04	0.27	0.91	0.50
Eubacterium sp CAG 251	4.1E-03	9.3E-03	0.34	0.01	0.24	6.5E-03	6.1E-03	0.33	0.72	0.49	5.1E-03	8.3E-03	0.36	0.13	0.37
Asaccharobacter celatus	4.3E-04	5.8E-04	0.58	0.01	0.24	5.0E-04	5.8E-04	0.57	0.54	0.49	2.7E-04	2.8E-04	0.43	0.25	0.38
Bacteroides salyersiae	2.5E-04	2.7E-04	0.16	0.01	0.24	6.8E-04	1.2E-03	0.18	0.61	0.49	5.8E-04	2.8E-04	0.18	0.19	0.37
Alistipes putredinis	5.9E-02	7.2E-02	0.98	0.02	0.24	5.4E-02	7.9E-02	0.98	0.00	0.08	6.5E-02	7.7E-02	0.98	0.11	0.37
Pseudonocardia ammonioxydans	3.8E-04	2.0E-04	0.42	0.02	0.24	1.8E-04	1.5E-04	0.30	0.57	0.49	1.1E-04	1.3E-04	0.28	0.08	0.34
Bacteroides sp 43 108	6.5E-05	5.0E-05	0.16	0.02	0.24	5.1E-05	5.5E-05	0.09	0.94	0.49	6.2E-05	6.9E-05	0.16	0.47	0.43
Barnesiella sp An22	9.9E-05	6.5E-05	0.26	0.02	0.24	7.6E-05	6.3E-05	0.23	0.29	0.41	4.9E-05	7.3E-05	0.20	0.05	0.34
Bacteroides ovatus	5.7E-03	9.0E-03	0.76	0.02	0.24	7.0E-03	1.2E-02	0.73	0.00	0.02	7.4E-03	1.4E-02	0.76	0.06	0.34
Alistipes indistinctus	9.8E-04	1.5E-03	0.43	0.02	0.26	1.5E-03	1.7E-03	0.44	0.01	0.10	1.9E-03	1.2E-03	0.43	0.17	0.37
Turicibacter sanguinis	6.3E-05	1.4E-04	0.15	0.02	0.26	2.1E-04	1.4E-04	0.21	0.20	0.36	1.9E-04	2.6E-04	0.20	0.72	0.48
Porphyromonas sp HMSC065F10	2.5E-04	1.4E-04	0.44	0.02	0.26	9.8E-05	2.2E-04	0.30	0.71	0.49	1.1E-04	1.1E-04	0.26	0.07	0.34
Blautia obeum	1.7E-03	1.9E-03	0.84	0.03	0.31	2.2E-03	2.4E-03	0.85	0.14	0.35	1.4E-03	1.6E-03	0.81	0.24	0.37
Prevotella stercorea	3.0E-02	1.4E-02	0.51	0.03	0.31	2.1E-02	1.3E-02	0.61	0.69	0.49	2.5E-02	1.6E-02	0.55	0.19	0.37
Phascolarctobacterium faecium	7.3E-03	9.8E-03	0.82	0.03	0.31	6.8E-03	9.3E-03	0.84	0.65	0.49	7.1E-03	6.9E-03	0.82	0.57	0.45
Bacteroidales bacterium KA00251	7.0E-05	1.1E-04	0.32	0.03	0.31	7.9E-05	9.6E-05	0.27	0.07	0.33	8.3E-05	1.0E-04	0.35	0.20	0.37
Dialister sp CAG 357	6.1E-03	1.6E-02	0.24	0.03	0.31	1.1E-02	1.4E-02	0.32	0.37	0.46	6.9E-03	1.8E-02	0.40	0.52	0.45
Firmicutes bacterium CAG 83	1.4E-03	2.3E-03	0.67	0.03	0.32	2.2E-03	2.4E-03	0.73	0.02	0.20	1.5E-03	1.7E-03	0.69	0.35	0.41
Prevotella sp AM42 24	1.2E-03	1.3E-04	0.54	0.04	0.32	7.8E-04	1.1E-04	0.50	0.19	0.36	1.2E-03	1.5E-04	0.52	0.78	0.49
Methanosphaera cuniculi	6.8E-05	4.0E-05	0.18	0.04	0.32	3.6E-05	4.9E-05	0.14	0.22	0.38	3.9E-05	8.6E-05	0.17	0.43	0.42

Table S2. The relative abundance and prevalence of microbial species included in the current analysis.

Species	T1					T2					T3				
	Mean case	Mean control	Prevalence	Wilcoxon P	Wilcoxon FDR	Mean case	Mean control	Prevalence	Wilcoxon P	Wilcoxon FDR	Mean case	Mean control	Prevalence	Wilcoxon P	Wilcoxon FDR
Alistipes shahii	2.5E-03	4.4E-03	0.74	0.04	0.32	3.5E-03	4.7E-03	0.74	0.06	0.31	3.9E-03	5.2E-03	0.77	0.15	0.37
Actinomyces massiliensis	5.8E-05	4.3E-05	0.18	0.04	0.33	4.0E-05	6.1E-05	0.16	0.84	0.49	5.5E-05	8.5E-05	0.23	0.24	0.37
Escherichia coli	1.5E-02	9.8E-03	0.93	0.04	0.33	2.1E-02	1.4E-02	0.91	0.03	0.20	1.6E-02	1.1E-02	0.82	0.61	0.47
Corynebacterium pyruviciproducens	7.0E-05	1.2E-04	0.23	0.04	0.33	8.1E-05	1.2E-04	0.23	0.84	0.49	1.0E-04	1.3E-04	0.25	0.83	0.49
Actinomyces sp oral taxon 414	7.5E-05	5.1E-05	0.17	0.05	0.33	7.2E-05	4.2E-05	0.17	0.06	0.31	7.5E-05	8.7E-05	0.17	0.58	0.46
Fusobacterium mortiferum	1.7E-03	5.5E-04	0.11	0.05	0.33	2.9E-03	3.4E-04	0.10	0.36	0.46	2.7E-03	2.1E-04	0.13	0.03	0.33
Corynebacterium pseudogenitalium	5.7E-05	7.0E-05	0.19	0.05	0.33	5.0E-05	5.7E-05	0.16	0.80	0.49	5.4E-05	8.2E-05	0.21	0.30	0.41
Firmicutes bacterium CAG 110	2.6E-04	6.8E-04	0.26	0.05	0.33	4.1E-04	7.3E-04	0.24	0.11	0.35	3.0E-04	3.3E-04	0.20	0.29	0.40
Fusicatenibacter saccharivorans	5.0E-03	6.9E-03	0.95	0.05	0.33	6.7E-03	6.4E-03	0.98	0.85	0.49	5.9E-03	4.6E-03	0.95	0.92	0.50
Prevotella disiens	1.3E-04	8.2E-05	0.39	0.05	0.34	1.1E-04	1.3E-04	0.37	0.15	0.35	1.8E-04	1.3E-04	0.48	0.15	0.37
Prevotella corporis	1.1E-04	9.6E-05	0.38	0.05	0.34	1.0E-04	1.2E-04	0.36	0.85	0.49	9.4E-05	9.2E-05	0.33	0.55	0.45
Streptococcus infantis	7.4E-05	3.9E-05	0.12	0.06	0.34	7.3E-05	6.1E-05	0.13	0.67	0.49	8.7E-05	2.9E-05	0.12	0.35	0.41
Proteobacteria bacterium CAG 139	3.4E-03	3.1E-03	0.54	0.06	0.34	4.4E-03	3.8E-03	0.54	0.08	0.35	3.4E-03	4.6E-03	0.57	0.49	0.44
Veillonella dispar	4.4E-04	3.1E-04	0.33	0.06	0.34	3.9E-04	6.8E-04	0.30	0.36	0.46	7.2E-04	3.6E-04	0.27	0.67	0.48
Tropheryma whipplei	2.7E-04	2.3E-04	0.39	0.06	0.34	3.2E-04	1.7E-04	0.32	0.00	0.08	1.4E-04	1.7E-04	0.33	0.07	0.34
Pseudomonas antarctica	6.3E-04	7.2E-04	0.35	0.06	0.34	6.8E-04	4.7E-04	0.48	0.15	0.35	5.6E-04	6.2E-04	0.40	0.85	0.50
Prevotella colorans	2.9E-04	1.9E-04	0.67	0.06	0.34	2.2E-04	1.8E-04	0.63	0.51	0.49	1.9E-04	3.0E-04	0.66	0.02	0.33
Corynebacterium singulare	1.2E-04	8.9E-05	0.37	0.06	0.34	8.6E-05	1.1E-04	0.33	0.69	0.49	1.0E-04	1.2E-04	0.35	0.65	0.48
Bacteroides stercoris	1.9E-02	2.8E-02	0.94	0.07	0.36	2.2E-02	2.9E-02	0.96	0.24	0.38	2.4E-02	4.1E-02	0.93	0.36	0.41
Eubacterium eligens	4.4E-02	5.0E-02	0.96	0.07	0.36	3.7E-02	4.9E-02	0.98	0.42	0.47	4.1E-02	6.2E-02	0.97	0.05	0.34
Gardnerella vaginalis	2.1E-04	2.9E-04	0.43	0.14	0.37	1.7E-04	1.2E-04	0.38	0.61	0.49	2.0E-04	2.3E-04	0.41	0.94	0.50
Bacteroides eggerthii	3.6E-03	8.6E-03	0.32	0.15	0.38	2.1E-03	1.3E-02	0.36	0.10	0.35	7.1E-03	1.0E-02	0.37	0.65	0.48
Haemophilus sp HMSC71H05	4.5E-04	1.1E-04	0.24	0.15	0.38	2.1E-04	1.2E-04	0.21	0.47	0.49	1.9E-04	3.9E-04	0.21	0.09	0.34
Clostridium sp CAG 58	1.2E-03	1.5E-03	0.42	0.16	0.39	1.3E-03	2.6E-03	0.44	0.00	0.01	1.0E-03	1.4E-03	0.38	0.26	0.38
Holdemania filiformis	1.2E-04	3.0E-04	0.28	0.17	0.40	2.2E-04	1.6E-04	0.26	0.80	0.49	2.1E-04	1.2E-04	0.23	0.13	0.37
Porphyromonas gingivalis	9.6E-05	1.2E-04	0.29	0.17	0.40	1.0E-04	9.3E-05	0.28	0.68	0.49	9.9E-05	1.0E-04	0.33	0.47	0.43
Bacteroides xylanisolvens	6.1E-03	1.1E-03	0.38	0.18	0.41	2.5E-03	1.1E-03	0.42	0.95	0.49	1.8E-03	1.3E-03	0.38	0.36	0.41
Cutibacterium granulosum	1.7E-04	1.3E-04	0.56	0.18	0.41	1.3E-04	1.4E-04	0.46	0.24	0.38	1.3E-04	1.1E-04	0.46	0.47	0.43
Actinomyces sp HMSC035G02	5.8E-05	3.9E-05	0.13	0.19	0.41	4.6E-05	3.4E-05	0.08	0.71	0.49	3.3E-05	4.5E-05	0.11	0.53	0.45

Table S2. The relative abundance and prevalence of microbial species included in the current analysis.

Species	T1					T2					T3				
	Mean case	Mean control	Prevalence	Wilcoxon P	Wilcoxon FDR	Mean case	Mean control	Prevalence	Wilcoxon P	Wilcoxon FDR	Mean case	Mean control	Prevalence	Wilcoxon P	Wilcoxon FDR
Clavibacter michiganensis	5.4E-05	4.0E-05	0.11	0.20	0.44	3.6E-05	3.7E-05	0.09	0.25	0.38	4.0E-05	5.7E-05	0.12	0.21	0.37
Haemophilus parainfluenzae	1.9E-03	8.4E-04	0.62	0.21	0.45	1.7E-03	7.0E-04	0.50	0.73	0.49	9.4E-04	9.2E-04	0.48	0.98	0.50
Prevotella pallens	9.9E-05	7.9E-05	0.26	0.22	0.45	7.9E-05	9.1E-05	0.26	0.40	0.46	8.2E-05	9.4E-05	0.23	0.30	0.41
Prevotella sp CAG 485	1.4E-04	1.3E-04	0.38	0.22	0.45	1.3E-04	1.1E-04	0.33	0.16	0.35	1.3E-04	1.0E-04	0.35	0.02	0.33
Enorma massiliensis	1.7E-04	1.6E-04	0.16	0.23	0.45	6.4E-05	2.5E-04	0.11	0.72	0.49	4.5E-05	1.4E-04	0.09	0.41	0.42
Clostridium clostridioforme	5.4E-04	2.9E-04	0.16	0.23	0.45	9.6E-05	8.5E-04	0.09	0.84	0.49	8.0E-05	8.1E-04	0.07	0.97	0.50
Bacteroides cellulosilyticus	3.7E-03	4.0E-03	0.49	0.25	0.45	2.9E-03	3.3E-03	0.47	0.24	0.38	1.5E-03	5.2E-03	0.48	0.08	0.34
Gordonibacter pamelaeae	2.6E-04	1.9E-04	0.40	0.25	0.45	3.8E-04	3.1E-04	0.39	0.45	0.48	3.5E-04	2.1E-04	0.39	0.32	0.41
Bacteroides sp OM05 12	3.5E-04	7.8E-04	0.30	0.25	0.45	3.3E-04	5.1E-04	0.28	0.81	0.49	3.6E-04	6.2E-04	0.29	0.74	0.48
Turicimonas muris	3.1E-04	2.8E-04	0.42	0.25	0.45	4.6E-04	3.0E-04	0.34	0.35	0.46	4.0E-04	4.1E-04	0.38	0.95	0.50
Prevotella sp CAG 520	4.4E-03	2.3E-03	0.43	0.26	0.45	9.0E-04	9.3E-04	0.36	0.71	0.49	5.6E-03	2.7E-03	0.48	0.89	0.50
Dialister invisus	1.2E-03	7.9E-04	0.17	0.26	0.45	6.8E-04	2.0E-04	0.10	0.48	0.49	2.7E-04	4.5E-04	0.10	0.15	0.37
Paraprevotella xylaniphila	1.0E-03	7.3E-04	0.32	0.27	0.45	1.4E-03	1.0E-03	0.37	0.63	0.49	9.8E-04	1.5E-03	0.33	0.43	0.42
Oscillibacter sp CAG 241	1.4E-03	1.2E-03	0.46	0.27	0.45	1.4E-03	1.7E-03	0.48	0.00	0.10	1.8E-03	8.8E-04	0.42	0.44	0.43
Roseburia sp CAG 471	1.2E-04	2.0E-04	0.23	0.27	0.45	1.5E-04	1.6E-04	0.20	0.55	0.49	8.2E-05	7.2E-05	0.15	0.74	0.48
Rhodococcus kroppenstedtii	1.1E-04	9.7E-05	0.36	0.27	0.45	1.0E-04	9.0E-05	0.32	0.86	0.49	1.2E-04	8.7E-05	0.26	0.47	0.43
Bilophila wadsworthia	1.3E-03	1.4E-03	0.76	0.27	0.45	2.1E-03	2.2E-03	0.81	0.11	0.35	2.0E-03	2.3E-03	0.82	0.68	0.48
Roseburia hominis	1.0E-02	1.2E-02	0.90	0.27	0.45	1.4E-02	1.1E-02	0.90	0.30	0.41	8.0E-03	7.9E-03	0.89	0.46	0.43
Corynebacterium variabile	1.3E-04	9.5E-05	0.27	0.27	0.45	1.1E-04	1.2E-04	0.33	0.70	0.49	9.0E-05	8.4E-05	0.24	0.56	0.45
Holdemanella biformis	2.1E-03	2.4E-03	0.20	0.28	0.45	2.1E-03	1.7E-03	0.22	0.39	0.46	1.7E-03	2.1E-03	0.20	0.14	0.37
Cutibacterium acnes	1.8E-04	2.8E-04	0.46	0.29	0.45	1.1E-04	9.7E-05	0.26	0.41	0.47	3.0E-04	2.3E-04	0.41	0.52	0.45
Eubacterium ramulus	1.4E-03	1.1E-03	0.80	0.29	0.45	2.6E-03	1.2E-03	0.83	0.01	0.12	2.0E-03	1.6E-03	0.83	0.17	0.37
Rothia aeria	7.4E-05	6.6E-05	0.16	0.29	0.45	6.0E-05	7.1E-05	0.16	0.99	0.50	6.2E-05	7.3E-05	0.18	0.24	0.37
Streptococcus oralis	1.1E-04	7.7E-05	0.20	0.29	0.45	1.4E-04	9.2E-05	0.21	0.19	0.36	1.2E-04	5.3E-05	0.16	0.54	0.45
Eubacterium ventriosum	3.8E-03	2.2E-03	0.69	0.30	0.46	3.9E-03	2.8E-03	0.74	0.53	0.49	2.1E-03	1.7E-03	0.63	0.99	0.50
Coprobacter secundus	1.4E-04	1.0E-04	0.35	0.32	0.46	1.0E-04	1.5E-04	0.30	0.16	0.35	9.4E-05	2.3E-04	0.30	0.31	0.41
Bacteroides fragilis	9.6E-04	1.7E-03	0.78	0.32	0.46	5.0E-04	2.6E-03	0.75	0.42	0.47	9.8E-04	4.6E-04	0.80	0.90	0.50
Streptococcus sanguinis	4.2E-05	3.5E-05	0.11	0.32	0.46	6.6E-05	1.3E-04	0.17	0.77	0.49	5.6E-05	7.6E-05	0.14	0.43	0.43
Actinomyces sp ICM47	1.8E-04	1.4E-04	0.32	0.32	0.46	1.2E-04	1.4E-04	0.29	0.99	0.50	2.0E-04	2.2E-04	0.39	0.65	0.48

Table S2. The relative abundance and prevalence of microbial species included in the current analysis.

Species	T1					T2					T3				
	Mean case	Mean control	Prevalence	Wilcoxon P	Wilcoxon FDR	Mean case	Mean control	Prevalence	Wilcoxon P	Wilcoxon FDR	Mean case	Mean control	Prevalence	Wilcoxon P	Wilcoxon FDR
Prevotella sp 885	8.0E-04	7.2E-05	0.17	0.32	0.46	6.8E-05	6.6E-05	0.18	0.25	0.38	8.4E-05	8.9E-05	0.19	0.42	0.42
Atopobium rimae	1.9E-04	8.9E-05	0.21	0.33	0.46	7.6E-05	8.3E-05	0.14	0.71	0.49	8.5E-05	9.2E-05	0.18	0.44	0.43
Parabacteroides johnsonii	7.3E-04	7.1E-04	0.15	0.33	0.46	7.1E-04	5.6E-04	0.15	0.15	0.35	5.6E-04	3.8E-04	0.14	0.33	0.41
Prevotella sp CAG 1185	1.4E-04	9.0E-05	0.31	0.33	0.46	9.0E-05	7.2E-05	0.25	0.12	0.35	9.1E-05	1.2E-04	0.33	0.11	0.37
Bacteroides intestinalis	1.9E-03	1.4E-03	0.36	0.33	0.46	1.9E-03	1.2E-03	0.30	0.69	0.49	1.8E-03	6.5E-04	0.33	0.62	0.47
Bacteroides uniformis	9.6E-03	1.9E-02	0.27	0.34	0.46	2.2E-02	1.9E-02	0.31	0.37	0.46	2.5E-02	1.1E-02	0.29	0.65	0.48
Oxalobacter formigenes	2.7E-04	2.7E-04	0.22	0.34	0.46	4.7E-04	2.9E-04	0.20	0.04	0.21	3.0E-04	1.2E-04	0.12	0.34	0.41
Slackia exigua	6.9E-05	5.3E-05	0.16	0.34	0.46	5.2E-05	7.0E-05	0.10	0.91	0.49	5.4E-05	7.2E-05	0.17	0.16	0.37
Odoribacter splanchnicus	4.5E-03	4.6E-03	0.78	0.34	0.46	4.4E-03	5.5E-03	0.77	0.23	0.38	6.0E-03	6.3E-03	0.80	1.00	0.50
Hungatella hathewayi	9.5E-04	4.4E-04	0.30	0.35	0.46	3.1E-04	3.6E-04	0.25	0.78	0.49	1.8E-04	3.7E-04	0.21	0.09	0.34
Roseburia sp CAG 182	6.5E-04	2.0E-03	0.14	0.36	0.46	5.4E-04	4.4E-04	0.17	0.79	0.49	3.5E-04	3.8E-04	0.12	0.55	0.45
Monoglobus pectinilyticus	1.0E-04	3.8E-04	0.12	0.39	0.46	4.3E-04	2.1E-04	0.10	0.93	0.49	5.6E-04	2.1E-04	0.11	0.99	0.50
Streptococcus vestibularis	3.8E-05	4.0E-05	0.09	0.39	0.46	1.2E-04	1.5E-04	0.22	0.77	0.49	2.0E-04	1.4E-04	0.24	0.67	0.48
Parabacteroides merdae	9.8E-03	1.2E-02	0.80	0.40	0.46	1.1E-02	1.5E-02	0.86	0.03	0.20	1.0E-02	1.6E-02	0.79	0.17	0.37
Streptococcus parasanguinis	2.2E-03	2.1E-03	0.85	0.40	0.46	2.4E-03	3.0E-03	0.88	0.54	0.49	2.4E-03	2.4E-03	0.86	0.36	0.41
Prevotella intermedia	6.0E-05	4.9E-05	0.20	0.41	0.46	4.9E-05	3.8E-05	0.12	0.19	0.36	4.6E-05	6.6E-05	0.15	0.93	0.50
Porphyromonas asaccharolytica	1.2E-04	1.8E-04	0.31	0.42	0.46	8.2E-05	1.0E-04	0.30	0.68	0.49	1.2E-04	1.0E-04	0.32	0.31	0.41
Actinomyces turicensis	2.4E-04	2.2E-04	0.50	0.42	0.46	1.8E-04	2.4E-04	0.50	0.81	0.49	2.9E-04	3.0E-04	0.52	0.17	0.37
Micrococcus luteus	1.3E-04	9.3E-05	0.37	0.42	0.46	7.5E-05	9.7E-05	0.27	0.95	0.49	8.6E-05	7.1E-05	0.26	0.23	0.37
Prevotella sp CAG 5226	7.9E-05	6.1E-05	0.18	0.42	0.46	6.0E-05	5.2E-04	0.20	0.03	0.21	3.5E-04	3.9E-04	0.21	0.63	0.48
Brevibacterium linens	1.2E-04	9.9E-05	0.36	0.42	0.46	8.9E-05	9.0E-05	0.34	0.52	0.49	9.3E-05	1.1E-04	0.33	0.67	0.48
Dysgonamonadaceae bacterium	6.4E-05	9.8E-05	0.14	0.43	0.46	5.5E-05	3.6E-05	0.09	0.06	0.31	5.8E-05	3.2E-05	0.14	0.15	0.37
Clostridium sp CAG 299	1.0E-03	7.0E-04	0.32	0.43	0.46	1.7E-03	2.0E-03	0.38	0.27	0.39	1.2E-03	4.1E-04	0.33	0.00	0.14
Eubacterium siraeum	1.4E-02	1.7E-02	0.61	0.43	0.46	1.3E-02	1.1E-02	0.59	0.85	0.49	1.2E-02	1.0E-02	0.49	0.04	0.34
Intestinimonas butyriciproducens	5.1E-04	4.5E-04	0.38	0.44	0.46	6.1E-04	3.7E-04	0.39	0.34	0.45	7.9E-04	2.6E-04	0.29	0.92	0.50
Eubacterium hallii	3.4E-03	3.0E-03	0.93	0.45	0.46	3.2E-03	3.2E-03	0.95	0.97	0.49	2.8E-03	2.6E-03	0.91	0.81	0.49
Aeriscardovia aeriphila	1.5E-04	1.8E-04	0.36	0.45	0.46	1.2E-04	1.4E-04	0.27	0.40	0.46	1.4E-04	1.4E-04	0.32	0.72	0.48
Gemella sanguinis	1.4E-04	8.1E-05	0.22	0.45	0.46	1.2E-04	9.2E-05	0.25	0.31	0.41	1.3E-04	1.2E-04	0.23	0.41	0.42
Olsenella uli	8.8E-05	6.2E-05	0.14	0.45	0.46	6.6E-05	7.2E-05	0.13	0.43	0.48	5.9E-05	8.2E-05	0.18	0.06	0.34

Table S2. The relative abundance and prevalence of microbial species included in the current analysis.

Species	T1					T2					T3				
	Mean case	Mean control	Prevalence	Wilcoxon P	Wilcoxon FDR	Mean case	Mean control	Prevalence	Wilcoxon P	Wilcoxon FDR	Mean case	Mean control	Prevalence	Wilcoxon P	Wilcoxon FDR
Dietzia alimentaria	1.1E-04	7.4E-05	0.16	0.45	0.46	8.3E-05	6.3E-05	0.17	0.98	0.50	1.1E-04	1.5E-04	0.23	0.40	0.42
Prevotella sp CAG 279	4.9E-03	3.1E-03	0.29	0.46	0.46	4.3E-03	2.9E-03	0.40	0.92	0.49	3.1E-03	6.2E-03	0.43	0.63	0.48
Delftia tsuruhatensis	9.8E-05	7.6E-05	0.16	0.46	0.46	1.1E-04	8.6E-05	0.13	0.61	0.49	1.7E-04	1.2E-04	0.23	0.95	0.50
Prevotella histicola	4.5E-05	5.5E-05	0.16	0.46	0.46	6.4E-05	3.3E-05	0.14	0.19	0.36	5.1E-05	1.2E-04	0.13	0.93	0.50
Parabacteroides goldsteinii	9.2E-04	5.6E-04	0.33	0.46	0.46	1.3E-03	5.5E-04	0.31	0.30	0.41	7.7E-04	4.9E-04	0.32	0.49	0.44
Prevotella sp CAG 617	1.3E-03	5.2E-04	0.19	0.46	0.46	1.8E-03	4.9E-04	0.19	0.16	0.35	8.6E-04	7.2E-04	0.16	0.39	0.42
Allisonella histaminiformans	5.2E-05	4.4E-05	0.09	0.47	0.46	1.1E-04	4.6E-05	0.11	0.88	0.49	7.5E-05	6.7E-05	0.11	0.42	0.42
Ruminococcus lactaris	6.5E-03	6.1E-03	0.62	0.47	0.46	7.3E-03	6.7E-03	0.62	0.76	0.49	5.9E-03	5.5E-03	0.63	0.27	0.39
Bacteroides massiliensis	7.5E-04	2.4E-04	0.41	0.47	0.46	8.7E-04	2.2E-04	0.39	1.00	0.50	5.7E-04	4.3E-04	0.40	0.03	0.34
Atopobium parvulum	1.7E-04	1.3E-04	0.41	0.47	0.46	9.0E-05	1.2E-04	0.30	0.87	0.49	9.2E-05	9.4E-05	0.27	0.51	0.45
Porphyromonas uenonis	2.8E-04	1.5E-04	0.48	0.48	0.46	1.9E-04	1.4E-04	0.45	0.03	0.20	1.5E-04	2.0E-04	0.41	0.56	0.45
Actinomyces sp oral taxon 897	7.3E-05	1.3E-04	0.19	0.48	0.46	1.4E-04	1.4E-04	0.20	0.83	0.49	7.7E-05	1.5E-04	0.19	0.77	0.49
Bifidobacterium dentium	1.4E-04	2.3E-04	0.34	0.48	0.46	1.0E-04	1.2E-04	0.28	0.19	0.36	1.4E-04	3.4E-04	0.44	0.03	0.34
Clostridium symbiosum	7.7E-04	4.4E-04	0.29	0.48	0.46	3.5E-04	3.7E-04	0.21	0.95	0.49	3.3E-04	1.8E-04	0.13	0.01	0.15
Roseburia faecis	2.8E-02	3.0E-02	0.96	0.48	0.46	2.9E-02	3.6E-02	0.97	0.22	0.38	2.1E-02	2.5E-02	0.94	0.25	0.38
Corynebacterium xerosis	1.7E-04	1.7E-04	0.39	0.48	0.46	1.0E-04	1.2E-04	0.31	0.13	0.35	9.7E-05	1.4E-04	0.32	0.04	0.34
Rothia mucilaginosa	2.0E-04	1.7E-04	0.74	0.50	0.46	1.7E-04	2.0E-04	0.67	0.42	0.47	3.3E-04	1.8E-04	0.70	0.85	0.50
Veillonella parvula	5.9E-03	3.4E-03	0.73	0.51	0.46	3.6E-03	1.7E-03	0.67	0.10	0.35	4.3E-03	3.0E-03	0.70	0.24	0.37
Prevotella amnii	3.3E-05	6.0E-05	0.10	0.51	0.46	5.2E-05	1.2E-04	0.17	0.23	0.38	7.4E-05	7.6E-05	0.14	0.84	0.50
Sanguibacter keddieii	5.8E-05	4.5E-05	0.13	0.52	0.46	5.6E-05	4.6E-05	0.14	0.67	0.49	5.4E-05	6.3E-05	0.18	0.26	0.38
Clostridium leptum	2.2E-04	7.5E-04	0.25	0.52	0.46	1.9E-04	5.5E-04	0.28	0.12	0.35	1.9E-04	2.2E-04	0.18	0.13	0.37
Collinsella aerofaciens	5.4E-03	4.3E-03	0.77	0.52	0.46	6.7E-03	5.9E-03	0.83	0.63	0.49	7.6E-03	5.6E-03	0.84	0.43	0.42
Lactobacillus rogosae	3.2E-04	1.7E-04	0.26	0.52	0.46	1.2E-04	2.0E-04	0.25	0.88	0.49	1.2E-04	1.3E-04	0.24	0.37	0.42
Tyzzerella nexilis	2.8E-04	1.4E-04	0.14	0.52	0.46	1.3E-03	4.2E-04	0.12	0.08	0.35	4.4E-04	4.6E-04	0.13	0.93	0.50
Rothia sp	3.3E-04	2.8E-04	0.38	0.52	0.46	1.1E-04	1.5E-04	0.28	0.93	0.49	8.0E-05	1.5E-04	0.28	0.01	0.15
Coprococcus eutactus	7.6E-03	8.5E-03	0.37	0.52	0.46	5.5E-03	9.3E-03	0.42	0.12	0.35	2.4E-03	5.5E-03	0.36	0.23	0.37
Neomicrococcus aestuarii	7.1E-05	5.7E-05	0.18	0.52	0.46	5.8E-05	4.3E-05	0.13	0.14	0.35	5.7E-05	7.2E-05	0.20	0.36	0.41
Clostridium bolteae CAG 59	1.6E-04	1.7E-04	0.16	0.53	0.46	8.4E-05	9.7E-05	0.15	0.19	0.36	1.2E-04	4.5E-05	0.09	0.60	0.47
Clostridium innocuum	3.0E-04	2.0E-04	0.22	0.53	0.46	2.3E-04	2.1E-04	0.19	0.59	0.49	1.4E-04	1.9E-04	0.15	0.03	0.33

Table S2. The relative abundance and prevalence of microbial species included in the current analysis.

Species	T1					T2					T3				
	Mean case	Mean control	Prevalence	Wilcoxon P	Wilcoxon FDR	Mean case	Mean control	Prevalence	Wilcoxon P	Wilcoxon FDR	Mean case	Mean control	Prevalence	Wilcoxon P	Wilcoxon FDR
Clostridium asparagiforme	8.4E-05	1.0E-04	0.15	0.54	0.46	3.8E-05	5.6E-05	0.12	0.84	0.49	7.5E-05	4.8E-05	0.10	0.76	0.49
Prevotella sp CAG 1320	1.5E-04	9.7E-05	0.32	0.54	0.46	8.8E-04	1.4E-04	0.30	0.26	0.39	9.0E-05	1.2E-04	0.32	0.34	0.41
Actinomyces meyeri	7.9E-05	6.9E-05	0.24	0.54	0.46	1.0E-04	1.2E-04	0.35	0.94	0.49	7.2E-05	8.1E-05	0.32	0.90	0.50
Corynebacterium durum	1.6E-04	3.1E-04	0.26	0.54	0.46	9.6E-05	9.4E-05	0.18	0.41	0.47	9.8E-05	2.9E-04	0.18	0.73	0.48
Janibacter hoylei	5.7E-05	5.6E-05	0.21	0.54	0.46	4.6E-05	5.4E-05	0.15	0.73	0.49	5.2E-05	6.9E-05	0.20	0.37	0.41
Actinomyces sp oral taxon 180	3.1E-05	2.6E-05	0.12	0.55	0.46	2.9E-05	2.7E-05	0.08	0.82	0.49	2.6E-05	4.8E-05	0.11	0.91	0.50
Bacteroides sp CAG 927	8.7E-05	6.8E-05	0.26	0.55	0.46	5.3E-05	1.8E-04	0.20	0.92	0.49	8.6E-05	1.0E-04	0.28	0.14	0.37
Prevotella buccae	6.4E-05	5.4E-05	0.16	0.55	0.46	4.6E-05	6.3E-05	0.16	0.61	0.49	6.2E-05	6.1E-05	0.20	0.60	0.47
Bacteroides finegoldii	2.9E-03	3.7E-03	0.54	0.56	0.46	2.8E-03	3.7E-03	0.60	0.45	0.48	7.0E-03	2.6E-03	0.58	0.94	0.50
Parabacteroides distasonis	1.3E-02	1.5E-02	0.97	0.56	0.46	1.4E-02	1.5E-02	0.97	0.14	0.35	1.7E-02	1.5E-02	0.98	0.08	0.34
Lachnospira pectinoschiza	2.8E-02	2.4E-02	0.81	0.56	0.46	4.0E-02	2.3E-02	0.79	0.78	0.49	2.7E-02	2.5E-02	0.82	0.93	0.50
Pseudarthrobacter phenanthrenivorans	1.0E-04	8.4E-05	0.23	0.56	0.46	9.8E-05	8.7E-05	0.24	0.53	0.49	8.6E-05	9.5E-05	0.24	0.06	0.34
Butyricimonas virosa	4.0E-04	3.0E-04	0.64	0.56	0.46	6.8E-04	7.2E-04	0.67	0.23	0.38	5.8E-04	4.3E-04	0.68	0.35	0.41
Prevotella copri	6.7E-02	5.1E-02	0.92	0.56	0.46	6.6E-02	5.7E-02	0.91	0.76	0.49	8.1E-02	9.7E-02	0.95	0.91	0.50
Megamonas hypermegale	1.6E-03	8.5E-04	0.32	0.57	0.46	2.2E-03	1.8E-03	0.40	0.16	0.35	1.1E-03	1.8E-03	0.40	0.23	0.37
Eubacterium sp OM08 24	2.0E-03	1.9E-03	0.20	0.58	0.46	3.6E-03	6.1E-04	0.15	0.68	0.49	8.7E-04	7.4E-04	0.24	0.78	0.49
Firmicutes bacterium CAG 170	3.3E-04	1.9E-04	0.17	0.58	0.46	3.7E-04	1.2E-03	0.18	0.79	0.49	2.4E-04	2.2E-04	0.13	0.91	0.50
Kocuria rosea	7.5E-05	7.1E-05	0.24	0.58	0.46	5.6E-05	5.7E-05	0.16	0.82	0.49	7.5E-05	7.1E-05	0.25	0.87	0.50
Veillonella infantium	2.1E-04	1.4E-04	0.26	0.59	0.46	1.9E-04	2.1E-04	0.21	0.16	0.35	4.4E-04	1.5E-04	0.19	0.70	0.48
Bacteroides galacturonicus	3.2E-04	3.1E-04	0.56	0.59	0.46	2.4E-04	2.0E-04	0.46	0.66	0.49	2.3E-04	2.7E-04	0.52	0.22	0.37
Actinomyces odontolyticus	1.1E-04	1.3E-04	0.21	0.59	0.46	1.6E-04	1.1E-04	0.16	0.94	0.49	1.1E-04	1.0E-04	0.19	0.50	0.44
Streptococcus anginosus group	4.0E-05	8.6E-05	0.11	0.62	0.47	9.4E-05	1.2E-04	0.18	0.28	0.40	4.5E-05	4.7E-05	0.12	0.89	0.50
Streptococcus mitis	1.0E-04	5.8E-05	0.16	0.62	0.47	1.2E-04	7.7E-05	0.17	0.13	0.35	6.2E-05	4.0E-05	0.16	0.17	0.37
Veillonella atypica	1.3E-03	1.3E-03	0.41	0.63	0.47	8.1E-04	1.3E-03	0.39	0.31	0.41	1.3E-03	1.1E-03	0.39	0.81	0.49
Akkermansia muciniphila	9.2E-03	7.1E-03	0.50	0.63	0.47	8.8E-03	5.3E-03	0.46	0.65	0.49	4.0E-03	2.5E-03	0.36	0.15	0.37
Actinomyces cardiffensis	2.1E-04	2.1E-04	0.54	0.64	0.47	2.1E-04	1.7E-04	0.55	0.03	0.20	2.0E-04	2.0E-04	0.55	0.81	0.49
Corynebacterium hadale	6.9E-05	6.7E-05	0.18	0.64	0.47	6.1E-05	6.5E-05	0.13	0.74	0.49	5.8E-05	6.8E-05	0.20	1.00	0.50
Micrococcus aloeverae	4.6E-04	4.2E-04	0.71	0.65	0.47	4.0E-04	5.0E-04	0.69	0.50	0.49	6.4E-04	4.6E-04	0.73	0.08	0.34

Table S2. The relative abundance and prevalence of microbial species included in the current analysis.

Species	T1					T2					T3				
	Mean case	Mean control	Prevalence	Wilcoxon P	Wilcoxon FDR	Mean case	Mean control	Prevalence	Wilcoxon P	Wilcoxon FDR	Mean case	Mean control	Prevalence	Wilcoxon P	Wilcoxon FDR
Clostridium bolteae	1.2E-03	1.2E-03	0.51	0.65	0.47	7.3E-04	1.5E-03	0.48	0.96	0.49	5.3E-04	4.7E-04	0.39	0.10	0.37
Lawsonella clevelandensis	8.4E-05	8.2E-05	0.29	0.65	0.47	6.4E-05	9.8E-05	0.24	0.09	0.35	8.5E-05	8.7E-05	0.30	0.68	0.48
Bacteroides caccae	5.4E-03	3.5E-03	0.35	0.65	0.47	5.6E-03	4.1E-03	0.34	0.56	0.49	7.8E-03	5.3E-03	0.36	0.37	0.41
Alistipes finegoldii	1.2E-02	8.7E-03	0.71	0.65	0.47	7.5E-03	8.3E-03	0.75	0.17	0.36	1.4E-02	6.6E-03	0.70	0.98	0.50
Roseburia sp CAG 303	7.0E-04	9.1E-04	0.33	0.65	0.47	1.2E-03	1.2E-03	0.30	0.93	0.49	1.1E-03	1.5E-03	0.30	0.68	0.48
Bifidobacterium adolescentis	4.2E-04	3.9E-04	0.39	0.66	0.47	4.3E-04	1.6E-03	0.33	0.14	0.35	4.9E-04	9.1E-04	0.38	0.52	0.45
Paraprevotella clara	4.6E-04	1.2E-04	0.29	0.66	0.47	3.5E-04	1.9E-04	0.31	0.61	0.49	8.9E-05	1.9E-04	0.34	0.12	0.37
Anaerotruncus colihominis	6.2E-04	1.3E-03	0.46	0.66	0.47	1.1E-03	9.6E-04	0.41	0.16	0.35	5.8E-04	3.8E-04	0.33	0.20	0.37
Methanobrevibacter arboriphilus	1.5E-04	1.3E-04	0.39	0.67	0.47	1.7E-04	1.4E-04	0.38	0.72	0.49	1.2E-04	1.2E-04	0.35	0.93	0.50
Collinsella intestinalis	1.8E-04	1.3E-04	0.25	0.67	0.47	1.5E-04	1.5E-04	0.18	0.90	0.49	1.5E-04	1.8E-04	0.17	0.01	0.15
Eubacterium rectale	6.5E-02	6.8E-02	0.96	0.68	0.47	5.4E-02	6.1E-02	0.98	0.65	0.49	5.0E-02	3.9E-02	0.98	0.77	0.49
Clostridium sp CAG 242	8.1E-04	1.0E-03	0.22	0.68	0.47	9.3E-04	5.5E-04	0.23	0.94	0.49	3.3E-04	4.2E-04	0.17	0.77	0.49
Cutibacterium avidum	5.3E-05	4.0E-05	0.11	0.68	0.47	3.2E-05	3.2E-05	0.07	0.89	0.49	4.0E-05	6.2E-05	0.15	0.07	0.34
Flavonifractor plautii	6.9E-03	5.1E-03	0.89	0.69	0.47	5.6E-03	3.9E-03	0.86	0.29	0.41	6.8E-03	5.6E-03	0.86	0.02	0.33
Bacteroides coprocola	3.6E-03	1.4E-03	0.21	0.69	0.47	1.7E-03	2.3E-03	0.20	0.39	0.46	2.0E-04	1.7E-03	0.21	0.06	0.34
Bacteroides sp D2	2.1E-04	1.9E-04	0.20	0.70	0.47	1.4E-04	1.5E-04	0.21	0.12	0.35	1.5E-04	3.3E-04	0.25	0.01	0.15
Oscillibacter sp 57 20	4.5E-03	4.8E-03	0.77	0.71	0.47	3.1E-03	4.9E-03	0.80	0.01	0.12	4.3E-03	4.0E-03	0.79	0.64	0.48
Streptococcus salivarius	5.4E-03	5.7E-03	0.98	0.71	0.47	7.4E-03	8.8E-03	1.00	0.51	0.49	7.7E-03	7.4E-03	0.97	1.00	0.50
Parascardovia denticolens	1.6E-04	1.6E-04	0.61	0.72	0.47	1.6E-04	1.3E-04	0.56	0.25	0.39	1.9E-04	1.7E-04	0.57	0.75	0.49
Butyricimonas synergistica	2.1E-04	2.4E-04	0.26	0.72	0.47	2.7E-04	2.8E-04	0.27	0.03	0.20	2.4E-04	1.9E-04	0.26	1.00	0.50
Coprococcus catus	9.2E-04	8.4E-04	0.66	0.72	0.47	1.4E-03	9.9E-04	0.68	0.74	0.49	1.3E-03	8.8E-04	0.70	0.23	0.37
Eubacterium sp CAG 274	5.6E-03	3.9E-03	0.61	0.72	0.47	1.0E-02	6.2E-03	0.66	0.39	0.46	6.3E-03	3.6E-03	0.61	0.07	0.34
Faecalibacterium prausnitzii	4.0E-02	3.9E-02	1.00	0.72	0.47	4.7E-02	3.9E-02	1.00	0.25	0.38	4.8E-02	4.0E-02	1.00	0.23	0.37
Roseburia intestinalis	5.6E-03	5.9E-03	0.72	0.72	0.47	5.6E-03	5.3E-03	0.73	0.81	0.49	3.4E-03	5.5E-03	0.70	0.45	0.43
Actinomyces sp oral taxon 448	1.8E-04	1.7E-04	0.50	0.73	0.47	1.5E-04	1.4E-04	0.50	0.42	0.47	1.6E-04	1.7E-04	0.51	0.94	0.50
Klebsiella quasipneumoniae	2.1E-04	3.8E-04	0.24	0.74	0.47	4.7E-04	5.1E-04	0.25	0.19	0.36	3.8E-04	2.3E-04	0.17	0.74	0.48
Roseburia inulinivorans	2.7E-02	2.6E-02	0.98	0.74	0.47	2.4E-02	1.4E-02	0.99	0.10	0.35	1.8E-02	1.5E-02	0.93	0.74	0.48
Eggerthella lenta	6.9E-04	8.9E-04	0.53	0.75	0.47	6.5E-04	2.2E-04	0.54	0.00	0.01	5.2E-04	3.6E-04	0.60	0.59	0.46
Desulfovibrio piger	2.3E-03	2.0E-03	0.21	0.75	0.47	2.3E-03	2.7E-03	0.20	0.80	0.49	1.8E-03	2.3E-03	0.19	0.95	0.50

Table S2. The relative abundance and prevalence of microbial species included in the current analysis.

Species	T1					T2					T3				
	Mean case	Mean control	Prevalence	Wilcoxon P	Wilcoxon FDR	Mean case	Mean control	Prevalence	Wilcoxon P	Wilcoxon FDR	Mean case	Mean control	Prevalence	Wilcoxon P	Wilcoxon FDR
Janibacter indicus	5.3E-05	4.1E-05	0.13	0.75	0.47	3.5E-05	3.6E-05	0.13	0.92	0.49	3.9E-05	4.4E-05	0.15	0.55	0.45
Bacteroides dorei	6.7E-03	1.6E-02	0.48	0.75	0.47	2.8E-03	1.0E-02	0.41	0.91	0.49	4.2E-03	3.3E-03	0.42	0.20	0.37
Eubacterium sp CAG 38	2.3E-02	1.5E-02	0.89	0.76	0.47	3.1E-02	1.2E-02	0.87	0.50	0.49	1.8E-02	1.4E-02	0.80	0.14	0.37
Corynebacterium efficiens	1.3E-04	1.1E-04	0.35	0.76	0.47	1.3E-04	1.5E-04	0.34	0.81	0.49	1.1E-04	9.6E-05	0.35	0.81	0.49
Veillonella sp T11011 6	3.4E-04	1.8E-04	0.23	0.77	0.47	2.7E-04	2.9E-04	0.23	0.69	0.49	3.0E-04	1.7E-04	0.22	0.66	0.48
Rothia dentocariosa	2.9E-04	2.0E-04	0.58	0.77	0.47	2.0E-04	1.9E-04	0.55	0.89	0.49	2.0E-04	1.7E-04	0.59	0.82	0.49
Bacteroides thetaiotaomicron	1.8E-02	1.8E-02	0.97	0.77	0.47	1.3E-02	1.7E-02	0.98	0.84	0.49	1.4E-02	1.5E-02	0.98	0.54	0.45
Agathobaculum butyriciproducens	3.6E-03	3.4E-03	0.94	0.77	0.47	3.9E-03	3.3E-03	0.95	0.27	0.40	3.6E-03	3.9E-03	0.91	0.93	0.50
Ruthenibacterium lactatiformans	3.5E-03	3.7E-03	0.87	0.78	0.47	6.3E-03	5.7E-03	0.86	0.46	0.49	4.1E-03	3.0E-03	0.80	0.28	0.40
Prevotella oris	4.0E-04	3.5E-04	0.68	0.78	0.47	3.6E-04	3.6E-04	0.65	0.82	0.49	5.2E-04	5.4E-04	0.72	0.56	0.45
Ruminococcus torques	8.5E-03	6.1E-03	0.79	0.79	0.47	9.1E-03	5.5E-03	0.86	0.56	0.49	1.1E-02	7.0E-03	0.87	0.24	0.37
Prevotella sp CAG 755	1.7E-04	1.5E-04	0.27	0.79	0.47	1.6E-04	1.1E-04	0.26	0.47	0.49	2.5E-04	2.2E-04	0.37	0.56	0.45
Prevotella buccalis	7.2E-05	5.7E-05	0.16	0.79	0.47	6.3E-05	7.4E-05	0.16	0.78	0.49	5.9E-05	6.9E-05	0.14	0.34	0.41
Parasutterella excrementihominis	3.6E-03	2.6E-03	0.67	0.79	0.47	4.9E-03	3.6E-03	0.63	0.77	0.49	5.6E-03	4.7E-03	0.64	0.13	0.37
Enterobacter cloacae complex	7.3E-04	8.3E-05	0.13	0.80	0.47	3.1E-04	1.7E-04	0.18	0.97	0.49	9.3E-05	1.5E-04	0.09	0.66	0.48
Streptococcus thermophilus	1.7E-03	1.4E-03	0.23	0.80	0.47	1.5E-03	9.5E-04	0.18	0.82	0.49	5.4E-04	1.6E-03	0.17	0.42	0.42
Clostridium citroniae	3.1E-04	2.9E-04	0.43	0.80	0.47	2.1E-04	2.7E-04	0.38	0.55	0.49	1.6E-04	1.8E-04	0.30	0.08	0.34
Bifidobacterium bifidum	2.5E-04	5.1E-04	0.25	0.81	0.47	1.6E-04	1.9E-04	0.34	0.72	0.49	1.6E-04	1.6E-04	0.27	0.38	0.42
Mycobacteroides abscessus	1.4E-04	1.5E-04	0.58	0.81	0.47	1.3E-04	1.6E-04	0.55	0.54	0.49	1.5E-04	1.8E-04	0.61	0.50	0.45
Bifidobacterium breve	1.4E-04	3.7E-04	0.13	0.82	0.47	3.1E-05	3.4E-05	0.08	0.93	0.49	6.6E-05	9.9E-05	0.11	0.21	0.37
Janibacter melonis	6.3E-05	6.5E-05	0.18	0.83	0.47	4.3E-05	4.8E-05	0.08	0.94	0.49	5.0E-05	6.1E-05	0.16	0.73	0.48
Dorea formicigenerans	3.2E-03	2.9E-03	0.92	0.83	0.47	3.6E-03	2.7E-03	0.94	0.37	0.46	2.6E-03	2.3E-03	0.92	0.27	0.39
Megamonas funiformis	1.3E-02	6.2E-03	0.46	0.83	0.47	8.1E-03	1.5E-02	0.65	0.23	0.38	7.6E-03	8.5E-03	0.58	0.98	0.50
Bacteroides coprophilus	5.3E-05	2.0E-04	0.10	0.83	0.47	1.6E-04	4.9E-05	0.09	0.79	0.49	7.8E-05	1.7E-04	0.14	0.36	0.41
Klebsiella variicola	1.1E-03	7.6E-04	0.34	0.84	0.48	1.3E-03	1.5E-03	0.36	0.40	0.46	9.6E-04	9.6E-04	0.23	0.96	0.50
Actinomyces georgiae	2.6E-04	3.0E-04	0.63	0.85	0.48	2.8E-04	3.8E-04	0.70	0.19	0.36	3.0E-04	3.2E-04	0.65	0.69	0.48
Sellimonas intestinalis	2.8E-04	3.9E-04	0.13	0.86	0.48	3.3E-04	5.5E-04	0.13	0.11	0.35	9.0E-05	2.1E-04	0.09	0.92	0.50
Ruminococcus gnavus	7.5E-03	3.3E-03	0.60	0.86	0.48	1.4E-02	5.1E-03	0.64	0.70	0.49	7.6E-03	4.4E-03	0.66	0.72	0.48
Sanguibacteroides justesenii	1.0E-04	4.7E-05	0.17	0.87	0.48	6.0E-05	7.5E-05	0.18	0.77	0.49	6.9E-05	6.0E-05	0.22	0.81	0.49

Table S2. The relative abundance and prevalence of microbial species included in the current analysis.

Species	T1					T2					T3				
	Mean case	Mean control	Prevalence	Wilcoxon P	Wilcoxon FDR	Mean case	Mean control	Prevalence	Wilcoxon P	Wilcoxon FDR	Mean case	Mean control	Prevalence	Wilcoxon P	Wilcoxon FDR
Lawsonibacter asaccharolyticus	8.7E-05	1.0E-04	0.23	0.87	0.48	9.5E-05	1.1E-04	0.23	0.99	0.50	2.7E-04	5.1E-05	0.15	0.06	0.34
Dorea longicatena	5.5E-03	5.0E-03	0.92	0.89	0.49	7.5E-03	5.5E-03	0.93	0.56	0.49	5.3E-03	8.3E-03	0.96	0.22	0.37
Bifidobacterium pseudocatenulatum	9.0E-03	6.0E-03	0.79	0.89	0.49	1.0E-02	1.2E-02	0.87	0.73	0.49	1.3E-02	1.2E-02	0.88	0.86	0.50
Bacteroides vulgatus	4.6E-02	4.3E-02	0.49	0.89	0.49	3.4E-02	2.2E-02	0.44	0.71	0.49	5.9E-02	2.3E-02	0.45	0.20	0.37
Coprobacter fastidiosus	8.6E-04	1.2E-03	0.40	0.92	0.49	6.4E-04	1.6E-03	0.33	0.87	0.49	6.0E-04	2.8E-03	0.36	0.17	0.37
Atopobium deltae	3.1E-04	2.8E-04	0.28	0.92	0.49	1.6E-04	1.2E-04	0.20	0.12	0.35	5.8E-04	2.9E-04	0.23	0.15	0.37
Eubacterium sp CAG 180	3.6E-02	3.7E-02	0.81	0.92	0.49	2.5E-02	2.7E-02	0.74	0.74	0.49	2.7E-02	3.1E-02	0.77	0.70	0.48
Blautia wexlerae	3.8E-03	4.2E-03	0.99	0.92	0.49	5.4E-03	4.9E-03	0.98	0.59	0.49	5.3E-03	2.5E-03	0.97	0.09	0.34
Bacteroides nordii	2.3E-04	9.0E-05	0.22	0.92	0.49	3.4E-04	7.2E-05	0.15	0.59	0.49	8.9E-05	1.2E-04	0.16	0.73	0.48
Bacteroides clarus	1.8E-03	1.1E-03	0.34	0.92	0.49	4.3E-04	5.9E-04	0.25	0.89	0.49	1.4E-03	5.2E-04	0.32	0.35	0.41
Corynebacterium kroppenstedtii	5.2E-05	5.0E-05	0.16	0.93	0.49	5.4E-05	4.4E-05	0.13	0.01	0.12	5.4E-05	5.7E-05	0.17	0.45	0.43
Corynebacterium matruchotii	4.1E-05	7.1E-05	0.13	0.93	0.49	1.3E-04	3.7E-05	0.12	0.07	0.33	5.8E-05	4.5E-05	0.11	0.78	0.49
Rhodococcus qingshengii	5.4E-05	5.5E-05	0.12	0.93	0.49	4.4E-05	5.1E-05	0.08	0.59	0.49	6.6E-05	5.7E-05	0.14	0.42	0.42
Bacteroides stercorirosoris	1.1E-04	6.8E-05	0.22	0.94	0.49	6.0E-05	5.9E-05	0.17	0.89	0.49	6.7E-05	7.1E-05	0.20	0.80	0.49
Ruminococcus callidus	2.0E-04	2.1E-04	0.18	0.94	0.49	1.5E-04	3.0E-04	0.18	0.39	0.46	3.0E-04	2.3E-04	0.21	0.97	0.50
Klebsiella pneumoniae	2.5E-03	2.6E-03	0.46	0.94	0.49	3.4E-03	4.7E-03	0.46	0.93	0.49	2.1E-03	2.7E-03	0.35	0.80	0.49
Gemmiger formicilis	4.3E-03	3.8E-03	0.80	0.95	0.49	4.9E-03	3.4E-03	0.84	0.68	0.49	3.2E-03	2.4E-03	0.85	0.69	0.48
Erysipelatoclostridium ramosum	1.9E-04	3.1E-04	0.15	0.95	0.49	7.3E-04	3.1E-04	0.14	0.91	0.49	2.0E-04	2.8E-04	0.13	0.34	0.41
Anaerostipes hadrus	1.0E-02	6.2E-03	0.97	0.96	0.49	1.2E-02	1.1E-02	0.98	0.02	0.17	1.3E-02	9.7E-03	0.95	0.11	0.37
Bacteroides sp CAG 144	6.1E-05	9.3E-05	0.17	0.96	0.49	4.8E-05	4.5E-05	0.13	0.29	0.41	5.0E-05	9.6E-05	0.19	0.53	0.45
Delftia acidovorans	6.0E-04	5.0E-04	0.42	0.97	0.49	6.1E-04	5.5E-04	0.47	0.56	0.49	1.0E-03	7.0E-04	0.65	0.89	0.50
Coprococcus comes	5.3E-03	5.5E-03	0.82	0.97	0.49	7.6E-03	7.6E-03	0.87	0.98	0.50	8.5E-03	5.9E-03	0.88	0.24	0.37
Micrococcus lylae	5.9E-05	5.5E-05	0.13	0.98	0.50	4.7E-05	4.6E-05	0.09	0.79	0.49	5.1E-05	5.8E-05	0.16	0.29	0.40
Bifidobacterium animalis	1.1E-04	1.4E-04	0.13	0.99	0.50	1.5E-04	1.2E-04	0.15	0.03	0.20	1.2E-04	2.1E-04	0.12	0.53	0.45
Actinomyces graevenitzii	2.1E-04	1.0E-04	0.20	0.99	0.50	1.4E-04	3.9E-05	0.17	0.01	0.13	7.0E-05	5.8E-05	0.15	0.41	0.42
Barnesiella intestinihominis	1.8E-03	3.8E-03	0.43	1.00	0.50	2.7E-03	5.8E-03	0.43	0.29	0.41	3.4E-03	2.6E-03	0.41	0.90	0.50
Prevotella sp CAG 1058	1.7E-04	9.4E-05	0.26	1.00	0.50	9.8E-05	1.4E-04	0.25	0.72	0.49	9.4E-05	2.9E-04	0.35	0.21	0.37

Table S3. The microbial pathways included in the current analysis.

Pathway	Class	T1					T2					T3				
		Mean case	Mean control	Prevalence	Wilcoxon P	Wilcoxon FDR	Mean case	Mean control	Prevalence	Wilcoxon P	Wilcoxon FDR	Mean case	Mean control	Prevalence	Wilcoxon P	Wilcoxon FDR
ANAEROFRUCAT-PWY	fermentation	6.0E-03	6.5E-03	1.00	0.00	0.17	6.1E-03	6.3E-03	1.00	0.36	0.61	6.0E-03	6.1E-03	1.00	0.71	0.46
ANAGLYCOLYSIS-PWY	glycolysis	8.4E-03	9.2E-03	1.00	0.00	0.03	8.6E-03	8.9E-03	1.00	0.20	0.41	8.7E-03	8.8E-03	1.00	0.72	0.46
FUC-RHAMCAT-PWY	sugar derivatives degradation	1.2E-03	1.2E-03	1.00	0.89	0.49	1.2E-03	1.1E-03	1.00	0.09	0.33	1.2E-03	9.8E-04	0.99	0.00	0.16
GALACT-GLUCUROCAT-PWY	sugar derivatives degradation	1.6E-03	1.5E-03	1.00	0.90	0.49	1.7E-03	1.4E-03	1.00	0.00	0.09	1.5E-03	1.4E-03	1.00	0.18	0.30
GALACTUROCAT-PWY	sugar derivatives degradation	1.5E-03	1.4E-03	1.00	0.94	0.49	1.6E-03	1.3E-03	1.00	0.01	0.10	1.4E-03	1.3E-03	1.00	0.32	0.37
GLUCUROCAT-PWY	sugar derivatives degradation	1.7E-03	1.7E-03	1.00	0.45	0.47	1.8E-03	1.6E-03	1.00	0.00	0.08	1.7E-03	1.6E-03	1.00	0.20	0.30
HISTSYN-PWY	amino acids biosynthesis	1.0E-02	1.0E-02	1.00	0.48	0.47	9.6E-03	1.0E-02	1.00	0.08	0.31	1.0E-02	1.1E-02	1.00	0.05	0.30
MET-SAM-PWY	amino acids biosynthesis	2.2E-03	2.0E-03	1.00	0.48	0.47	2.4E-03	2.0E-03	1.00	0.01	0.13	2.2E-03	1.8E-03	1.00	0.06	0.30
P164-PWY	nucleosides and nucleotides degradation	9.9E-04	9.6E-04	1.00	0.68	0.49	1.1E-03	9.4E-04	1.00	0.00	0.08	1.1E-03	8.7E-04	0.97	0.00	0.16
PWY-1042	glycolysis	1.5E-02	1.5E-02	1.00	0.39	0.44	1.4E-02	1.5E-02	1.00	0.01	0.13	1.5E-02	1.5E-02	1.00	0.45	0.39
PWY-5177	carboxylates degradation	1.6E-03	1.5E-03	1.00	0.34	0.44	1.9E-03	1.5E-03	1.00	0.01	0.09	1.6E-03	1.4E-03	1.00	0.18	0.30
PWY-6113	fatty acid and lipid biosynthesis	1.1E-03	9.1E-04	0.82	0.19	0.40	1.2E-03	1.0E-03	0.88	0.04	0.21	1.1E-03	8.9E-04	0.78	0.11	0.30
PWY-6121	nucleosides and nucleotides biosynthesis	1.1E-02	1.1E-02	1.00	0.01	0.23	1.1E-02	1.1E-02	1.00	0.53	0.71	1.1E-02	1.1E-02	1.00	0.57	0.42
PWY-6353	nucleosides and nucleotides degradation	1.7E-03	1.7E-03	1.00	0.91	0.49	1.9E-03	1.6E-03	1.00	0.01	0.09	1.8E-03	1.5E-03	1.00	0.10	0.30
PWY-6608	nucleosides and nucleotides degradation	2.0E-03	1.8E-03	1.00	0.62	0.49	2.2E-03	1.7E-03	1.00	0.00	0.08	2.3E-03	1.7E-03	1.00	0.00	0.16
PWY-6609	nucleosides and nucleotides biosynthesis	1.4E-02	1.4E-02	1.00	0.60	0.49	1.3E-02	1.4E-02	1.00	0.01	0.10	1.4E-02	1.4E-02	1.00	0.16	0.30
PWY-6700	nucleosides and nucleotides biosynthesis	1.2E-02	1.2E-02	1.00	0.74	0.49	1.2E-02	1.3E-02	1.00	0.03	0.20	1.3E-02	1.3E-02	1.00	0.10	0.30
PWY-6703	secondary metabolites biosynthesis	7.5E-03	7.6E-03	1.00	0.98	0.50	7.4E-03	7.7E-03	1.00	0.33	0.58	7.7E-03	8.6E-03	1.00	0.02	0.30
PWY-7211	nucleosides and nucleotides biosynthesis	1.6E-03	1.7E-03	0.99	0.59	0.49	1.7E-03	1.4E-03	0.99	0.01	0.09	1.7E-03	1.5E-03	0.99	0.07	0.30
PWY-7237	sugar derivatives degradation	8.0E-03	7.1E-03	1.00	0.06	0.33	7.7E-03	7.0E-03	1.00	0.06	0.26	7.7E-03	7.4E-03	0.98	0.53	0.40
PWY-7242	sugar derivatives degradation	1.4E-03	1.4E-03	1.00	0.68	0.49	1.5E-03	1.3E-03	1.00	0.00	0.09	1.4E-03	1.3E-03	1.00	0.16	0.30
PWY-7456	polysaccharides degradation	2.1E-03	2.4E-03	0.99	0.00	0.17	2.1E-03	2.1E-03	1.00	0.65	0.79	2.1E-03	2.0E-03	1.00	0.63	0.43
PWY0-1319	fatty acid and lipid biosynthesis	1.1E-02	1.1E-02	1.00	0.01	0.23	1.1E-02	1.1E-02	1.00	0.27	0.50	1.1E-02	1.1E-02	1.00	0.53	0.40
PWY0-162	nucleosides and nucleotides biosynthesis	4.5E-03	4.7E-03	1.00	0.12	0.37	4.4E-03	4.8E-03	1.00	0.04	0.21	4.6E-03	5.0E-03	1.00	0.08	0.30
SALVADEHYPOX-PWY	nucleosides and nucleotides degradation	1.4E-03	1.2E-03	1.00	0.36	0.44	1.6E-03	1.2E-03	1.00	0.00	0.08	1.4E-03	1.2E-03	0.99	0.10	0.30
TRNA-CHARGING-PWY	aminoacyl-trnas charging	1.3E-02	1.3E-02	1.00	0.39	0.44	1.3E-02	1.3E-02	1.00	0.00	0.09	1.3E-02	1.3E-02	1.00	0.16	0.30
1CMET2-PWY	vitamin biosynthesis	1.1E-02	1.1E-02	1.00	0.07	0.33	1.1E-02	1.1E-02	1.00	0.09	0.31	1.1E-02	1.1E-02	1.00	0.41	0.38
ARG+POLYAMINE-SYN	amino acids biosynthesis	1.8E-03	1.7E-03	1.00	0.58	0.49	1.9E-03	1.7E-03	1.00	0.09	0.32	1.6E-03	1.5E-03	0.99	0.44	0.39
ARO-PWY	amino acids biosynthesis	1.1E-02	1.1E-02	1.00	0.14	0.37	1.1E-02	1.1E-02	1.00	0.82	0.91	1.1E-02	1.0E-02	1.00	0.62	0.43

Table S3. The microbial pathways included in the current analysis.

Pathway	Class	T1					T2					T3				
		Mean case	Mean control	Prevalence	Wilcoxon P	Wilcoxon FDR	Mean case	Mean control	Prevalence	Wilcoxon P	Wilcoxon FDR	Mean case	Mean control	Prevalence	Wilcoxon P	Wilcoxon FDR
ASPASN-PWY	amino acids biosynthesis	6.6E-03	6.3E-03	1.00	0.93	0.49	6.1E-03	6.5E-03	1.00	0.25	0.47	6.9E-03	7.2E-03	1.00	0.42	0.38
BIOTIN-BIOSYNTHESIS-PWY	vitamin biosynthesis	2.5E-03	2.4E-03	1.00	0.57	0.49	2.3E-03	2.2E-03	1.00	0.54	0.71	2.4E-03	2.3E-03	1.00	0.88	0.49
BRANCHED-CHAIN-AA-SYN-PWY	amino acids biosynthesis	1.2E-02	1.2E-02	1.00	0.59	0.49	1.1E-02	1.2E-02	1.00	0.02	0.19	1.2E-02	1.2E-02	1.00	0.47	0.39
CALVIN-PWY	carbohydrates biosynthesis	9.8E-03	1.0E-02	1.00	0.24	0.42	9.8E-03	1.0E-02	1.00	0.27	0.50	9.4E-03	9.3E-03	1.00	0.51	0.40
COA-PWY	cofactor biosynthesis	9.9E-03	1.0E-02	1.00	0.17	0.38	1.0E-02	1.0E-02	1.00	0.13	0.36	1.0E-02	1.0E-02	1.00	0.95	0.49
COBALSYN-PWY	vitamin biosynthesis	3.5E-03	3.6E-03	1.00	0.24	0.42	3.8E-03	3.5E-03	1.00	0.11	0.35	3.4E-03	3.3E-03	1.00	0.29	0.36
COLANSYN-PWY	carbohydrates biosynthesis	2.5E-03	2.7E-03	1.00	0.04	0.33	2.6E-03	2.4E-03	1.00	0.19	0.41	2.5E-03	2.3E-03	1.00	0.13	0.30
COMPLETE-ARO-PWY	amino acids biosynthesis	1.0E-02	1.1E-02	1.00	0.13	0.37	1.1E-02	1.1E-02	1.00	0.97	1.00	1.0E-02	1.0E-02	1.00	0.81	0.48
DAPLYSINESYN-PWY	amino acids biosynthesis	1.3E-03	1.2E-03	1.00	0.43	0.46	1.5E-03	1.4E-03	0.99	0.47	0.68	1.5E-03	1.3E-03	0.99	0.08	0.30
DTDPRHAMSYN-PWY	carbohydrates biosynthesis	1.7E-02	1.6E-02	1.00	0.44	0.46	1.5E-02	1.6E-02	1.00	0.33	0.58	1.6E-02	1.7E-02	1.00	0.15	0.30
FASYN-ELONG-PWY	fatty acid and lipid biosynthesis	2.6E-03	2.4E-03	1.00	0.27	0.42	2.5E-03	2.3E-03	1.00	0.45	0.67	2.5E-03	2.4E-03	1.00	0.79	0.48
FERMENTATION-PWY	fermentation	1.1E-03	1.0E-03	0.98	0.72	0.49	1.2E-03	1.1E-03	0.99	0.14	0.37	1.1E-03	9.3E-04	0.97	0.29	0.36
GLCMANNANAUT-PWY	amine and polyamine degradation	2.1E-03	2.1E-03	1.00	0.87	0.49	2.2E-03	1.9E-03	1.00	0.04	0.23	2.0E-03	1.8E-03	1.00	0.04	0.30
GLUCONEO-PWY	carbohydrates biosynthesis	4.1E-03	4.3E-03	1.00	0.10	0.37	4.1E-03	4.3E-03	1.00	0.13	0.36	4.1E-03	4.1E-03	1.00	0.88	0.49
GLUTORN-PWY	amino acids biosynthesis	8.1E-03	8.4E-03	1.00	0.44	0.46	8.5E-03	8.2E-03	1.00	0.41	0.65	7.7E-03	7.5E-03	1.00	0.72	0.46
GLYCOGENSYNTH-PWY	carbohydrates biosynthesis	5.7E-03	6.0E-03	1.00	0.29	0.43	6.3E-03	5.8E-03	1.00	0.07	0.28	5.7E-03	5.4E-03	1.00	0.15	0.30
GLYCOLYSIS-E-D	glycolysis	2.3E-03	2.4E-03	1.00	0.24	0.42	2.4E-03	2.4E-03	1.00	0.80	0.90	2.3E-03	2.2E-03	1.00	0.44	0.39
GLYCOLYSIS	glycolysis	4.9E-03	5.2E-03	1.00	0.26	0.42	5.1E-03	5.2E-03	1.00	0.38	0.63	5.0E-03	5.1E-03	1.00	0.51	0.40
HEXITOLDEGSUPER-PWY	sugar derivatives degradation	2.0E-03	1.9E-03	1.00	0.35	0.44	2.1E-03	2.0E-03	1.00	0.39	0.64	2.0E-03	1.8E-03	1.00	0.16	0.30
HISDEG-PWY	amino acids degradation	3.7E-03	3.9E-03	1.00	0.31	0.43	3.7E-03	3.7E-03	1.00	0.55	0.71	4.2E-03	4.2E-03	1.00	1.00	0.50
HSERMETANA-PWY	amino acids biosynthesis	5.5E-03	5.6E-03	1.00	0.33	0.43	5.9E-03	5.5E-03	1.00	0.06	0.26	5.5E-03	5.6E-03	1.00	0.69	0.46
ILEUSYN-PWY	amino acids biosynthesis	1.5E-02	1.5E-02	1.00	0.80	0.49	1.4E-02	1.5E-02	1.00	0.12	0.36	1.5E-02	1.5E-02	1.00	0.38	0.38
NAD-BIOSYNTHESIS-II	NAD metabolism	9.9E-04	8.6E-04	0.92	0.28	0.42	1.1E-03	8.6E-04	0.91	0.24	0.45	1.0E-03	1.0E-03	0.92	0.75	0.47
NAGLIPASYN-PWY	cell structures biosynthesis	2.1E-03	2.2E-03	1.00	0.37	0.44	2.1E-03	2.1E-03	1.00	0.80	0.90	2.3E-03	2.3E-03	1.00	0.97	0.49
NONOXIPENT-PWY	generation of precursor metabolite and energy	6.2E-03	6.1E-03	1.00	0.93	0.49	6.5E-03	6.2E-03	1.00	0.25	0.47	5.7E-03	5.4E-03	1.00	0.20	0.30
OANTIGEN-PWY	cell structures biosynthesis	5.7E-03	5.9E-03	1.00	0.38	0.44	6.1E-03	6.0E-03	1.00	0.56	0.71	5.7E-03	5.7E-03	1.00	0.76	0.47
P441-PWY	carboxylates degradation	1.6E-03	1.4E-03	1.00	0.27	0.42	1.7E-03	1.4E-03	0.99	0.06	0.27	1.4E-03	1.2E-03	0.99	0.32	0.37

Table S3. The microbial pathways included in the current analysis.

Pathway	Class	T1					T2					T3				
		Mean case	Mean control	Prevalence	Wilcoxon P	Wilcoxon FDR	Mean case	Mean control	Prevalence	Wilcoxon P	Wilcoxon FDR	Mean case	Mean control	Prevalence	Wilcoxon P	Wilcoxon FDR
PANTO-PWY	vitamin biosynthesis	9.4E-03	9.4E-03	1.00	0.63	0.49	9.1E-03	9.6E-03	1.00	0.04	0.21	9.5E-03	9.7E-03	1.00	0.35	0.38
PANTOSYN-PWY	cofactor biosynthesis	9.4E-03	9.5E-03	1.00	0.57	0.49	9.2E-03	9.7E-03	1.00	0.02	0.17	9.6E-03	9.7E-03	1.00	0.62	0.43
PENTOSE-P-PWY	generation of precursor metabolite and energy	3.3E-03	3.3E-03	1.00	0.75	0.49	3.3E-03	3.2E-03	1.00	0.98	1.00	3.2E-03	3.2E-03	1.00	0.71	0.46
PHOSLIPSYN-PWY	fatty acid and lipid biosynthesis	5.0E-03	5.0E-03	1.00	0.89	0.49	4.8E-03	4.9E-03	1.00	0.86	0.92	4.9E-03	4.8E-03	1.00	0.44	0.39
POLYISOPRENSYN-PWY	polyprenyl biosynthesis	1.5E-03	1.5E-03	1.00	0.72	0.49	1.5E-03	1.4E-03	1.00	0.47	0.68	1.6E-03	1.5E-03	1.00	0.82	0.48
PRPP-PWY	amino acids biosynthesis	2.5E-03	2.5E-03	1.00	0.75	0.49	2.7E-03	2.4E-03	1.00	0.04	0.21	2.5E-03	2.4E-03	1.00	0.32	0.37
PWY-1269	carbohydrates biosynthesis	3.8E-03	3.5E-03	1.00	0.69	0.49	3.3E-03	3.5E-03	1.00	0.50	0.70	3.9E-03	3.9E-03	1.00	0.97	0.49
PWY-2941	amino acids biosynthesis	2.7E-03	2.8E-03	1.00	0.56	0.49	2.9E-03	2.7E-03	1.00	0.38	0.63	2.9E-03	2.9E-03	1.00	0.92	0.49
PWY-2942	amino acids biosynthesis	1.1E-02	1.1E-02	1.00	0.65	0.49	1.1E-02	1.1E-02	1.00	0.07	0.28	1.1E-02	1.2E-02	1.00	0.10	0.30
PWY-3001	amino acids biosynthesis	8.7E-03	8.9E-03	1.00	0.22	0.42	8.5E-03	8.8E-03	1.00	0.13	0.36	8.7E-03	8.9E-03	1.00	0.18	0.30
PWY-4981	amino acids biosynthesis	2.1E-03	2.0E-03	1.00	0.77	0.49	2.1E-03	2.1E-03	1.00	0.97	1.00	2.3E-03	2.2E-03	1.00	0.93	0.49
PWY-4984	inorganic nutrients metabolism	2.1E-03	2.3E-03	0.99	0.33	0.43	2.1E-03	2.2E-03	1.00	0.69	0.81	2.3E-03	2.4E-03	1.00	0.50	0.40
PWY-5005	vitamin biosynthesis	1.1E-03	1.0E-03	0.92	0.63	0.49	1.1E-03	1.1E-03	0.92	0.41	0.65	9.6E-04	1.1E-03	0.92	0.49	0.40
PWY-5100	fermentation	4.6E-03	5.0E-03	1.00	0.10	0.37	4.9E-03	4.9E-03	1.00	0.62	0.77	4.4E-03	4.4E-03	1.00	0.93	0.49
PWY-5104	amino acids biosynthesis	2.1E-03	2.2E-03	1.00	1.00	0.50	2.3E-03	2.3E-03	1.00	0.30	0.53	2.1E-03	1.9E-03	1.00	0.04	0.30
PWY-5154	amino acids biosynthesis	6.5E-03	6.4E-03	1.00	0.97	0.50	5.9E-03	6.1E-03	1.00	0.49	0.69	6.0E-03	6.3E-03	1.00	0.25	0.35
PWY-5188	cofactor biosynthesis	2.9E-03	2.9E-03	1.00	0.95	0.49	3.2E-03	3.2E-03	1.00	0.78	0.89	2.8E-03	3.0E-03	1.00	0.42	0.38
PWY-5345	amino acids biosynthesis	1.4E-03	1.3E-03	0.97	0.31	0.43	1.5E-03	1.3E-03	0.97	0.22	0.44	1.3E-03	1.1E-03	0.92	0.50	0.40
PWY-5484	glycolysis	4.5E-03	4.8E-03	1.00	0.16	0.37	4.6E-03	4.7E-03	1.00	0.46	0.68	4.6E-03	4.7E-03	1.00	0.55	0.40
PWY-5505	amino acids biosynthesis	1.4E-03	1.4E-03	1.00	0.81	0.49	1.4E-03	1.3E-03	1.00	0.14	0.37	1.3E-03	1.3E-03	1.00	0.94	0.49
PWY-5659	carbohydrates biosynthesis	3.1E-03	3.2E-03	1.00	0.23	0.42	3.2E-03	3.0E-03	1.00	0.13	0.36	3.2E-03	3.0E-03	1.00	0.38	0.38
PWY-5686	nucleosides and nucleotides biosynthesis	1.5E-02	1.5E-02	1.00	0.77	0.49	1.4E-02	1.5E-02	1.00	0.15	0.37	1.5E-02	1.5E-02	1.00	0.27	0.36
PWY-5695	polyamine biosynthesis	1.2E-02	1.2E-02	1.00	0.86	0.49	1.1E-02	1.2E-02	1.00	0.15	0.37	1.2E-02	1.3E-02	1.00	0.08	0.30
PWY-5838	quinone biosynthesis	1.1E-03	9.7E-04	0.97	0.15	0.37	1.1E-03	9.5E-04	0.96	0.36	0.61	9.5E-04	9.4E-04	0.92	0.74	0.47
PWY-5941	polysaccharides degradation	1.3E-03	1.4E-03	0.97	0.11	0.37	1.1E-03	1.1E-03	0.93	0.96	1.00	1.3E-03	1.2E-03	0.97	0.88	0.49
PWY-5971	fatty acid and lipid biosynthesis	1.9E-03	1.6E-03	0.82	0.10	0.37	2.0E-03	1.7E-03	0.89	0.05	0.26	1.8E-03	1.5E-03	0.79	0.20	0.30
PWY-5973	fatty acid and lipid biosynthesis	8.2E-03	8.6E-03	1.00	0.23	0.42	7.9E-03	8.4E-03	1.00	0.16	0.38	8.5E-03	9.0E-03	1.00	0.11	0.30
PWY-6122	nucleosides and nucleotides biosynthesis	1.2E-02	1.2E-02	1.00	0.84	0.49	1.2E-02	1.2E-02	1.00	0.23	0.45	1.2E-02	1.3E-02	1.00	0.32	0.37

Table S3. The microbial pathways included in the current analysis.

Pathway	Class	T1					T2					T3				
		Mean case	Mean control	Prevalence	Wilcoxon P	Wilcoxon FDR	Mean case	Mean control	Prevalence	Wilcoxon P	Wilcoxon FDR	Mean case	Mean control	Prevalence	Wilcoxon P	Wilcoxon FDR
PWY-6123	nucleosides and nucleotides biosynthesis	7.6E-03	8.2E-03	1.00	0.08	0.33	7.8E-03	8.1E-03	1.00	0.45	0.67	8.1E-03	8.3E-03	1.00	0.60	0.43
PWY-6126	nucleosides and nucleotides biosynthesis	8.4E-03	8.7E-03	1.00	0.01	0.23	8.7E-03	8.5E-03	1.00	0.40	0.64	8.7E-03	8.6E-03	1.00	0.52	0.40
PWY-6147	vitamin biosynthesis	6.5E-03	6.2E-03	1.00	0.61	0.49	5.7E-03	6.3E-03	1.00	0.19	0.41	6.6E-03	7.0E-03	1.00	0.40	0.38
PWY-6151	amino acids biosynthesis	1.1E-02	1.1E-02	1.00	0.76	0.49	1.1E-02	1.1E-02	1.00	0.57	0.71	1.1E-02	1.2E-02	1.00	0.33	0.37
PWY-6163	aromatic compounds biosynthesis	1.2E-02	1.2E-02	1.00	0.21	0.42	1.2E-02	1.2E-02	1.00	0.54	0.71	1.2E-02	1.2E-02	1.00	0.87	0.49
PWY-621	aromatic compounds degradation	2.1E-03	2.2E-03	1.00	0.39	0.44	2.2E-03	2.2E-03	1.00	0.78	0.89	2.2E-03	2.0E-03	1.00	0.20	0.30
PWY-6317	carbohydrates degradation	5.8E-03	6.1E-03	1.00	0.21	0.42	6.0E-03	5.6E-03	1.00	0.18	0.41	5.4E-03	5.0E-03	1.00	0.18	0.30
PWY-6387	cell structures biosynthesis	1.4E-02	1.4E-02	1.00	0.82	0.49	1.3E-02	1.4E-02	1.00	0.02	0.15	1.4E-02	1.4E-02	1.00	0.22	0.32
PWY-6470	cell structures biosynthesis	1.4E-03	1.4E-03	0.98	0.57	0.49	1.5E-03	1.4E-03	0.98	0.59	0.73	1.5E-03	1.2E-03	0.97	0.13	0.30
PWY-6471	cell structures biosynthesis	2.1E-03	2.3E-03	0.99	0.54	0.49	2.4E-03	2.3E-03	0.98	0.49	0.69	2.3E-03	2.0E-03	0.97	0.09	0.30
PWY-6507	sugar derivatives degradation	1.4E-03	1.4E-03	1.00	0.84	0.49	1.5E-03	1.3E-03	1.00	0.10	0.33	1.3E-03	1.4E-03	1.00	0.49	0.40
PWY-6545	nucleosides and nucleotides biosynthesis	3.2E-03	3.2E-03	1.00	0.90	0.49	3.3E-03	3.3E-03	1.00	0.99	1.00	3.0E-03	3.2E-03	1.00	0.09	0.30
PWY-6737	polysaccharides degradation	1.4E-02	1.4E-02	1.00	0.01	0.23	1.4E-02	1.4E-02	1.00	0.90	0.95	1.3E-02	1.3E-02	1.00	0.38	0.38
PWY-6892	vitamin biosynthesis	4.6E-03	5.0E-03	1.00	0.08	0.33	4.6E-03	4.7E-03	1.00	0.50	0.70	4.8E-03	4.6E-03	1.00	0.39	0.38
PWY-6895	vitamin biosynthesis	2.0E-03	2.0E-03	0.99	0.90	0.49	2.0E-03	1.8E-03	0.99	0.09	0.31	1.8E-03	1.6E-03	0.95	0.17	0.30
PWY-6897	vitamin biosynthesis	7.1E-03	7.0E-03	1.00	0.77	0.49	6.6E-03	7.0E-03	1.00	0.17	0.39	7.3E-03	7.4E-03	1.00	0.23	0.33
PWY-6901	carbohydrates degradation	2.9E-03	3.1E-03	1.00	0.13	0.37	3.0E-03	3.0E-03	1.00	0.75	0.87	2.9E-03	2.9E-03	1.00	0.72	0.46
PWY-7184	nucleosides and nucleotides biosynthesis	4.2E-03	4.1E-03	1.00	0.99	0.50	4.2E-03	4.1E-03	1.00	0.56	0.71	4.1E-03	4.1E-03	1.00	0.84	0.49
PWY-7196	nucleosides and nucleotides biosynthesis	1.2E-03	1.2E-03	0.99	0.69	0.49	1.3E-03	1.1E-03	0.97	0.04	0.21	1.2E-03	1.1E-03	0.95	0.13	0.30
PWY-7197	nucleosides and nucleotides biosynthesis	2.8E-03	2.8E-03	1.00	0.95	0.49	2.9E-03	2.8E-03	1.00	0.69	0.81	2.7E-03	2.8E-03	1.00	0.73	0.46
PWY-7198	nucleosides and nucleotides biosynthesis	1.1E-03	1.1E-03	0.99	0.49	0.48	1.1E-03	1.1E-03	0.96	0.85	0.92	1.0E-03	1.2E-03	0.98	0.58	0.42
PWY-7199	nucleosides and nucleotides biosynthesis	9.0E-03	9.0E-03	1.00	0.95	0.49	8.6E-03	9.1E-03	1.00	0.05	0.24	9.2E-03	9.4E-03	1.00	0.37	0.38
PWY-7208	nucleosides and nucleotides biosynthesis	4.9E-03	4.7E-03	1.00	0.42	0.46	4.9E-03	4.6E-03	1.00	0.20	0.41	4.7E-03	4.9E-03	1.00	0.46	0.39
PWY-7219	nucleosides and nucleotides biosynthesis	1.5E-02	1.5E-02	1.00	0.95	0.49	1.4E-02	1.5E-02	1.00	0.03	0.21	1.5E-02	1.5E-02	1.00	0.19	0.30
PWY-7220	nucleosides and nucleotides biosynthesis	6.6E-03	6.7E-03	1.00	0.81	0.49	7.2E-03	6.7E-03	1.00	0.14	0.37	7.0E-03	6.8E-03	1.00	0.34	0.37
PWY-7221	nucleosides and nucleotides biosynthesis	1.4E-02	1.4E-02	1.00	0.44	0.46	1.3E-02	1.4E-02	1.00	0.02	0.14	1.4E-02	1.4E-02	1.00	0.16	0.30
PWY-7228	nucleosides and nucleotides biosynthesis	6.3E-03	6.1E-03	1.00	0.56	0.49	6.4E-03	6.0E-03	1.00	0.19	0.41	6.6E-03	6.7E-03	1.00	0.76	0.47
PWY-7229	nucleosides and nucleotides biosynthesis	9.3E-03	9.6E-03	1.00	0.01	0.23	9.5E-03	9.4E-03	1.00	0.86	0.92	9.6E-03	9.6E-03	1.00	0.92	0.49
PWY-7234	nucleosides and nucleotides biosynthesis	2.3E-03	2.4E-03	1.00	0.28	0.42	2.4E-03	2.7E-03	1.00	0.15	0.37	2.5E-03	2.6E-03	1.00	0.73	0.46

Table S3. The microbial pathways included in the current analysis.

Pathway	Class	T1					T2					T3				
		Mean case	Mean control	Prevalence	Wilcoxon P	Wilcoxon FDR	Mean case	Mean control	Prevalence	Wilcoxon P	Wilcoxon FDR	Mean case	Mean control	Prevalence	Wilcoxon P	Wilcoxon FDR
PWY-7323	carbohydrates biosynthesis	2.4E-03	2.6E-03	1.00	0.08	0.33	2.4E-03	2.3E-03	1.00	0.23	0.45	2.4E-03	2.3E-03	1.00	0.79	0.48
PWY-7400	amino acids biosynthesis	9.3E-03	9.7E-03	1.00	0.28	0.42	9.7E-03	9.5E-03	1.00	0.56	0.71	8.8E-03	8.7E-03	1.00	0.83	0.49
PWY-7663	fatty acid and lipid biosynthesis	7.6E-03	7.8E-03	1.00	0.30	0.43	7.3E-03	7.7E-03	1.00	0.20	0.41	7.8E-03	8.2E-03	1.00	0.16	0.30
PWY-841	nucleosides and nucleotides biosynthesis	6.9E-03	7.0E-03	1.00	0.72	0.49	6.9E-03	6.8E-03	1.00	0.45	0.67	6.9E-03	7.2E-03	1.00	0.33	0.37
PWY0-1061	amino acids biosynthesis	1.5E-03	1.6E-03	0.95	0.83	0.49	1.4E-03	1.5E-03	0.92	0.99	1.00	1.3E-03	1.1E-03	0.92	0.90	0.49
PWY0-1261	sugar derivatives degradation	3.4E-03	3.4E-03	1.00	0.85	0.49	3.5E-03	3.4E-03	1.00	0.74	0.86	3.6E-03	3.4E-03	1.00	0.41	0.38
PWY0-1296	nucleosides and nucleotides degradation	7.4E-03	7.8E-03	1.00	0.16	0.37	7.7E-03	7.5E-03	1.00	0.42	0.65	7.1E-03	6.7E-03	1.00	0.19	0.30
PWY0-1297	nucleosides and nucleotides degradation	9.6E-04	8.4E-04	0.99	0.79	0.49	1.2E-03	9.4E-04	0.98	0.06	0.26	9.9E-04	8.9E-04	0.95	0.27	0.36
PWY0-1479	nucleosides and nucleotides biosynthesis	1.5E-03	1.5E-03	1.00	0.99	0.50	1.5E-03	1.4E-03	1.00	0.51	0.70	1.3E-03	1.4E-03	1.00	0.32	0.37
PWY0-1586	cell structures biosynthesis	1.0E-02	9.8E-03	1.00	0.47	0.47	1.0E-02	1.0E-02	1.00	0.83	0.92	1.0E-02	1.1E-02	1.00	0.92	0.49
PWY0-166	nucleosides and nucleotides biosynthesis	4.9E-03	4.9E-03	1.00	0.84	0.49	5.0E-03	5.0E-03	1.00	0.89	0.94	4.9E-03	4.9E-03	1.00	0.97	0.49
PWY0-781	amino acids biosynthesis	1.1E-03	1.0E-03	0.99	0.32	0.43	1.3E-03	1.1E-03	0.98	0.13	0.36	1.2E-03	1.0E-03	0.97	0.08	0.30
PWY0-845	vitamin biosynthesis	2.9E-03	3.2E-03	1.00	0.16	0.37	2.9E-03	3.1E-03	1.00	0.43	0.67	3.3E-03	3.1E-03	1.00	0.95	0.49
PWY4FS-7	fatty acid and lipid biosynthesis	3.8E-03	3.7E-03	1.00	0.86	0.49	3.7E-03	3.6E-03	1.00	0.63	0.77	3.7E-03	3.5E-03	1.00	0.38	0.38
PYRIDNUCSAL-PWY	NAD metabolism	1.1E-03	1.0E-03	0.96	0.80	0.49	1.3E-03	1.1E-03	0.96	0.11	0.35	1.2E-03	1.0E-03	0.92	0.39	0.38
PYRIDNUCSYN-PWY	NAD metabolism	8.0E-03	8.0E-03	1.00	0.91	0.49	7.7E-03	8.1E-03	1.00	0.11	0.35	8.2E-03	8.5E-03	1.00	0.14	0.30
RHAMCAT-PWY	carbohydrates degradation	4.1E-03	3.9E-03	1.00	0.75	0.49	3.6E-03	3.8E-03	1.00	0.18	0.40	4.0E-03	4.0E-03	1.00	0.94	0.49
RIBOSYN2-PWY	vitamin biosynthesis	1.0E-02	1.0E-02	1.00	0.93	0.49	9.8E-03	1.0E-02	1.00	0.08	0.31	1.0E-02	1.1E-02	1.00	0.09	0.30
SER-GLYSYN-PWY	amino acids biosynthesis	6.4E-03	6.7E-03	1.00	0.16	0.37	6.6E-03	6.7E-03	1.00	0.45	0.67	6.6E-03	6.8E-03	1.00	0.29	0.36
TCA	glycolysis	1.5E-03	1.5E-03	1.00	0.66	0.49	1.7E-03	1.6E-03	1.00	0.16	0.37	1.5E-03	1.3E-03	1.00	0.47	0.39
TEICHOICACID-PWY	cell structures biosynthesis	1.1E-03	1.2E-03	1.00	0.14	0.37	1.2E-03	1.2E-03	1.00	1.00	1.00	1.2E-03	1.2E-03	1.00	0.97	0.49
THISYN-PWY	vitamin biosynthesis	6.4E-03	6.7E-03	1.00	0.11	0.37	6.1E-03	6.4E-03	1.00	0.12	0.36	6.5E-03	6.8E-03	1.00	0.18	0.30
THRESYN-PWY	amino acids biosynthesis	7.7E-03	7.9E-03	1.00	0.55	0.49	7.6E-03	7.7E-03	1.00	0.54	0.71	7.8E-03	8.0E-03	1.00	0.28	0.36
TRPSYN-PWY	amino acids biosynthesis	6.4E-03	6.6E-03	1.00	0.11	0.37	6.6E-03	6.5E-03	1.00	0.86	0.92	6.3E-03	6.5E-03	1.00	0.54	0.40
UDPNAGSYN-PWY	cell structures biosynthesis	4.1E-03	4.1E-03	1.00	0.66	0.49	4.6E-03	4.5E-03	1.00	0.69	0.81	4.2E-03	4.2E-03	1.00	0.62	0.43

Table S4. Baseline characteristics of the included and excluded GDM cases.

	Included n=120	Excluded n=298	*P*-value
**Age, years**	27.5 (±4.0)	28.2 (±4.2)	0.12
**Smoking history**			0.64
Current/Former	5 (4.2)	18 (6.0)	
Never	115 (95.8)	280 (94.0)	
**Drinking history**			0.95
Current/Former	23 (19.1)	59 (19.8)	
Never	97 (80.8)	239 (80.2)	
**Education**			0.88
Junior secondary	40 (33.3)	93 (31.2)	
Senior secondary	62 (51.7)	157 (52.7)	
Tertiary	18 (15.0)	48 (16.1)	
**Family history of type 2 diabetes**	6 (5.0)	31 (10.4)	0.088
**Parity**			0.42
0	71 (59.2)	155 (52.0)	
>1	49 (40.8)	143 (48.0)	
**Prepregnancy BMI, kg/m^2**	21.6 (±3.1)	22.2 (±3.3)	0.062
**Baseline BMI, kg/m^2**	22.0 (±3.0)	22.6 (±3.3)	0.083
**Gestational weight gain, kg**	1.2 (±2.8)	1.1 (±3.3)	0.76
**Systolic blood pressure, mmHg**	111.6 (±10.2)	111.5 (±10.1)	0.96
**Diastolic blood pressure, mmHg**	74.5 (±8.5)	74.3 (±8.7)	0.8
**Physical activity levels, METs**	116.1 (±65.0)	129.7 (±77.8)	0.17
**Fasting plasma glucose, mmol/L**	4.5 (±0.5)	4.5 (±0.5)	0.35
**Hemoglobin A1c, %**	5.2 (±0.3)	5.2 (±0.3)	0.69

Data were shown as Mean ± SD for continuous variables or N (%) for categorical variables. The group comparisons were conducted using Student's t-test or Wilcox ranked sum test for continuous variables and $\chi 2$ test or Fisher's exact test for categorical variables.
